# Genome-wide analyses identify 60 loci associated with liver triglyceride content

**DOI:** 10.1038/s41588-026-02794-6

**Published:** 2026-10-21

**Authors:** Oveis Jamialahmadi, Shahir Mowlaei, Kyla Z. Gelev, Rosellina M. Mancina, Valentina Flagiello, Julia Kozlitina, Päivi Pajukanta, Luca Valenti, Stefano Romeo

**Affiliations:** 1 Centre for Reproduction, Metabolism and Molecular Medicine (CeRM), Department of Medicine, Karolinska Institute, Huddinge, Sweden; 2 Department of Human Genetics, David Geffen School of Medicine at UCLA, Los Angeles, CA USA; 3 Department of Life Science, Health, and Health Professions, Link Campus University, Rome, Italy; 4 Operative Research Unit of Clinical Medicine and Hepatology, Fondazione Policlinico Universitario Campus Bio-Medico, Rome, Italy; 5 The Eugene McDermott Center for Human Growth and Development, University of Texas Southwestern Medical Center, Dallas, TX USA; 6 Bioinformatics Interdepartmental Program, UCLA, Los Angeles, CA USA; 7 Institute for Precision Health, David Geffen School of Medicine at UCLA, Los Angeles, CA USA; 8 Department of Pathophysiology and Transplantation, Università degli Studi di Milano, Milan, Italy; 9 Precision Medicine and Biological Resource Center, Fondazione IRCCS Ca’ Granda Ospedale Maggiore Policlinico Milano, Milan, Italy; 10 Department of Endocrinology, Karolinska University Hospital, Huddinge, Sweden; 11 Department of Medical and Surgical Sciences, Magna Graecia University, Catanzaro, Italy; 12 Department of Cardiology, Sahlgrenska University Hospital, Gothenburg, Sweden; 13 Department of Molecular and Clinical Medicine, University of Gothenburg, Gothenburg, Sweden

**Keywords:** Genetics research, Genome-wide association studies

## Abstract

We performed a large-scale genome-wide association study meta-analysis of magnetic resonance imaging-derived liver triglyceride content across 71,488 European-ancestry participants from the UK Biobank, approximately doubling the sample size of previous genome-wide association studies^1^. We identified 60 independent loci, including 31 not previously reported, and estimated the single nucleotide polymorphism-based heritability of liver triglyceride content at 25%. Integrative fine-mapping and gene-prioritization analyses nominated candidate effector genes for 48 loci. Of the 31 newly identified variants, 14 were validated in meta-analyses of clinically diagnosed metabolic dysfunction-associated steatotic liver disease (42,163 cases and 1,646,254 controls). People in the highest versus lowest decile of a polygenic risk score for liver triglyceride content had 2.71-fold higher odds of cirrhosis and 7.47-fold higher odds of hepatocellular carcinoma. Plasma proteins associated with the liver triglyceride polygenic risk score were enriched for metabolic remodeling and inflammatory pathways, with liver-enriched protein signatures increasing and visceral adipose signatures decreasing with higher genetic risk. Together, these findings refine the genetic architecture of liver triglyceride content, linking polygenic susceptibility to downstream metabolic and inflammatory pathways and providing insights into the genetic basis of steatotic liver disease.

## Abstract (web summary)

Genome-wide association analyses of liver triglyceride content in 71,488 UK Biobank participants identify 60 independent loci and yield polygenic scores correlated with higher risk of cirrhosis and hepatocellular carcinoma.

## Main

Steatotic liver disease (SLD) is a complex trait defined by excessive fat accumulation within hepatocytes, mainly as triglycerides. SLD has a strong genetic component, with most of the common variants increasing the susceptibility to higher liver triglyceride content^2^. The noninvasive gold standard for liver triglyceride content measurement is magnetic resonance imaging (MRI)^3^, which is an expensive and not widely available technique. Therefore, the number of participants with available liver triglyceride measurements pooled in genome-wide studies examining the genetic contribution to liver steatosis is still limited^4,5^, hampering the discovery of a larger fraction of its heritability.

In this study, we performed a large-scale genome-wide association study (GWAS) of liver triglyceride content (*n* = 71,488), identifying 60 loci and estimating the single nucleotide polymorphism (SNP)-based heritability of liver triglyceride content at 25%. We combined three nonoverlapping MRI-derived datasets, including one generated in the present study by extracting proton density fat fraction (PDFF) from 42,028 iterative decomposition of water and fat with echo asymmetry and least-squares estimation (IDEAL) liver acquisitions. We next prioritized candidate effector genes for the associated loci by integrating fine-mapping with complementary gene-prioritization approaches and liver *cis*-expression quantitative trait loci (*cis*-eQTL) colocalization. We then validated the previously unreported variants against clinically diagnosed metabolic dysfunction-associated steatotic liver disease (MASLD) (42,163 cases and 1,646,254 controls), assessed their associations with cirrhosis and hepatocellular carcinoma (HCC) and used polygenic risk scores (PRSs) to examine the downstream clinical and molecular consequences of inherited susceptibility to liver triglyceride content, including phenome-wide and plasma proteomic analyses.

We extracted and estimated MRI-derived PDFF from 42,028 IDEAL acquisitions in the UK Biobank (Methods). We removed 188 scans that showed potential anomalies when compared with the fat-referenced IDEAL PDFF^6^ (Supplementary Fig. 1 and Supplementary Table 1). Our PDFF estimation showed near-perfect agreement with 1,550 Perspectum-derived values^7^ (*r* = 0.99) and with 15,135 Advanced MR Analytics (AMRA)-derived fat-referenced IDEAL PDFF values^6^ (*r* = 0.98). Next, we performed genome-wide association analyses of liver triglyceride content in 71,488 European-ancestry participants using three nonoverlapping PDFF datasets: gradient-echo PDFF^8^ (*n* = 1,200), IDEAL-derived PDFF measured in this study (*n* = 35,179) and Perspectum-derived PDFF^7^ (*n* = 35,109). Owing to different imaging protocols and PDFF derivation, we performed a separate GWAS for each dataset and combined the resulting summary statistics through a fixed-effect inverse variance-weighted meta-analysis. We also repeated this association analysis after harmonizing the three PDFF measures to a common scale to verify that the findings did not depend on the underlying PDFF definition (Supplementary Fig. 2). We used linkage disequilibrium score regression (LDSC^9^) to assess confounding and SNP-based heritability. The LDSC intercept showed no evidence of inflation (0.99, s.e. = 0.052), and the SNP-based heritability of PDFF was 25% (s.e. = 0.077).

**Fig. 1:**
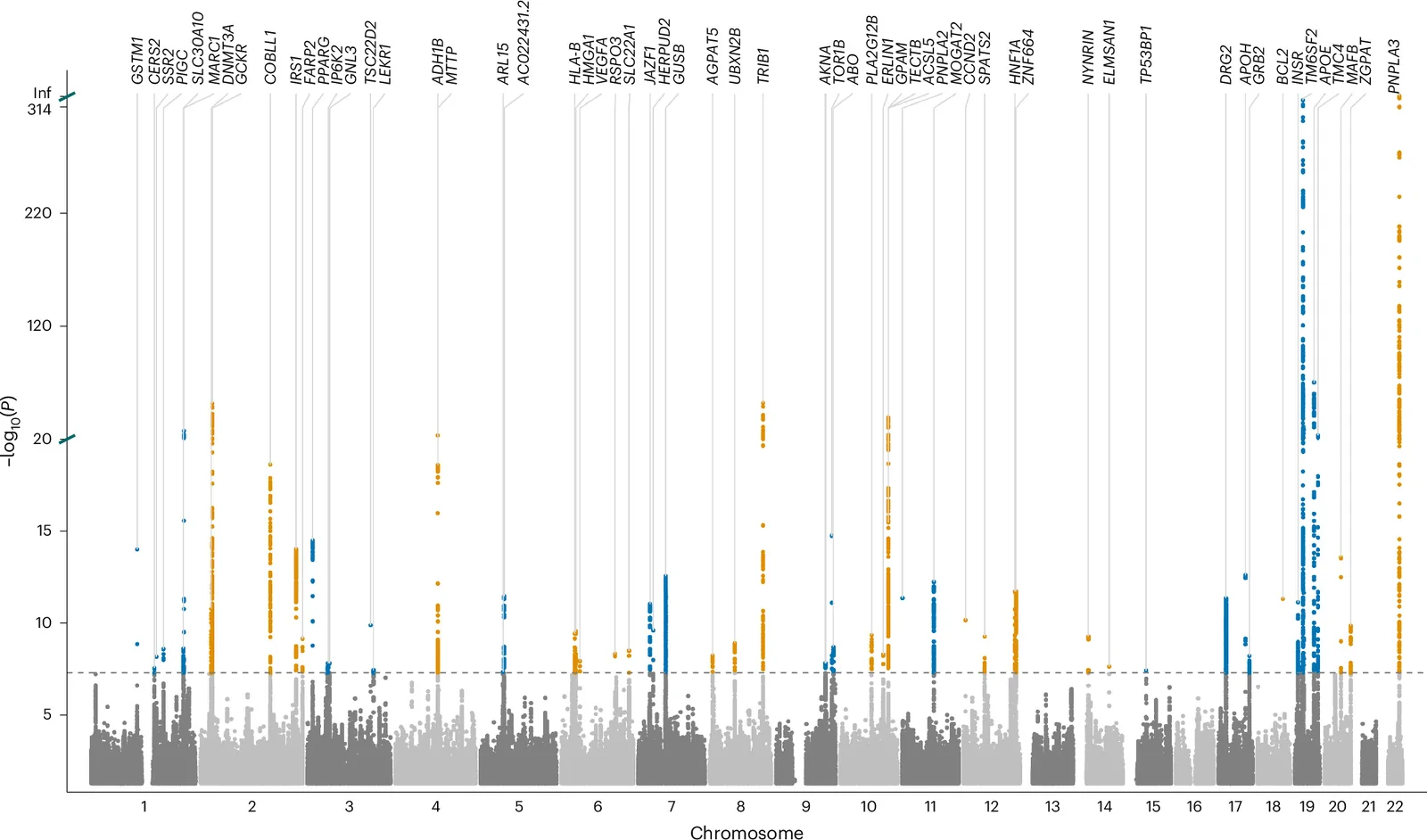
Manhattan plot of the GWAS meta-analysis of liver triglyceride content in 71,488 European participants from the UK Biobank. The gray dashed line shows the genome-wide significance threshold (5 × 10^−8^). Blue and orange dots indicate genome-wide significant associations, with colors alternating between chromosomes. Gray dots indicate associations that did not reach this threshold. The *x* axis shows the chromosome numbers and positions. The *y* axis shows two-sided −log_10_(*P* value). Inf denotes infinity and indicates *P* values below machine precision. Association analyses for the three PDFF datasets were performed using a whole-genome regression model implemented in REGENIE, adjusting for age, sex, age × sex, age^2^, age^2^ × sex, BMI, the first ten genomic PCs and array batch. Summary statistics from the three analyses were then combined using a fixed-effect inverse variance-weighted meta-analysis. *P* values were two-sided and not corrected for multiple testing.

To identify genome-wide significant signals, we defined approximately independent loci through linkage disequilibrium (LD) clumping and conditional analyses, and fine-mapped all loci exceeding genome-wide significance (Methods). In total, we identified 60 independent genome-wide significant associations for PDFF across fine-mapped regions and regions that were not included in fine-mapping (Fig. 1 and Supplementary Tables 2 and 3). Independent lead variants at 12 loci (*GSTM1*, *MARC1*, *GCKR*, *TSC22D2*, *ADH1B*, *TRIB1*, *TOR1B*, *PNPLA2*, *CCND2*, *BCL2*, *TM6SF2* and *APOE*) showed posterior inclusion probability values of ≥0.95, suggesting that they are putative causal variants. When we compared these findings with our recent multi-adiposity-adjusted GWAS of PDFF^1^ including 36,394 participants, 31 of the 60 independent associations were not previously reported (Supplementary Table 2). Previous evidence for these loci is summarized in the Supplementary Note. Additional adjustment for alcohol intake produced effects highly concordant with the primary GWAS (Supplementary Note and Supplementary Table 4).

We next prioritized candidate effector genes for the 60 independent loci identified in our fine-mapping analysis. We integrated Fine-Mapped Locus Assessment Model of Effector Genes (FLAMES)^10^ and Calling Disease-Related Genes (CALDERA)^11^ gene-prioritization evidence with liver *cis*-eQTL colocalization (Methods). For each locus, we assigned binary confidence scores for the three approaches and summed them to obtain an overall confidence score. The only locus where we prioritized colocalization over FLAMES^10^ or CALDERA^11^ was the *TMC4* locus, where colocalization supported *MBOAT7*, consistent with previous functional evidence supporting *MBOAT7* at this locus^12,13^. Overall, 48 loci had a confidence score above 1, and 37 of the prioritized candidate genes were also the nearest genes. FLAMES prioritized *CYP7A1* at the *UBXN2B* locus. We recently showed that this locus interacts with body mass index (BMI) for circulating alanine aminotransferase (ALT) levels in European participants from the UK Biobank, and in vitro and in vivo studies confirmed *CYP7A1* as the causal gene at this locus^14^. Prioritized effector genes and their confidence scores are reported in Supplementary Table 5.

To validate the associations for variants not previously reported, we meta-analyzed GWAS summary statistics for clinically diagnosed MASLD (42,163 cases and 1,646,254 controls) across five studies of European ancestry (deCODE, Intermountain, FinnGen, Million Veteran Program (MVP) and UK Biobank^15–18^; Supplementary Tables 6a and 7). In the UK Biobank, we excluded participants with available PDFF measurements (*n* = 71,488) before the meta-analysis. To assess the genome-wide genetic overlap between MRI-derived liver triglyceride content and clinically diagnosed MASLD, we estimated their genetic correlation using bivariate LDSC. PDFF and MASLD showed a strong genetic correlation (*r*_g_ = 0.74, s.e. = 0.08, *P* = 2.5 × 10^−21^), supporting the use of MASLD as a validation phenotype for PDFF loci. Of the 31 genetic variants, 14 (45%) reached Bonferroni significance (*P* < 1.6 × 10^−3^) and 22 (71%) were nominally significant (*P* < 0.05), all with concordant directions of effect compared with the discovery analyses (Supplementary Table 6a). The 14 Bonferroni-significant loci were *CERS2*, *SLC30A10*, *FARP2*, *LEKR1*, *ARL15*, *HERPUD2*, *AKNA*, *ERLIN1*, *ACSL5*, *CCND2*, *HNF1A*, *TP53BP1*, *INSR* and *ZGPAT*; among the remaining 17 loci, 8 reached nominal significance.

In addition, we examined the association of variants not previously reported with cirrhosis and HCC. The variants at the *GRB2* and *ERLIN1* loci were associated with cirrhosis and HCC, respectively, after correction for multiple testing (Supplementary Note and Supplementary Tables 6b and 6c).

**Fig. 2:**
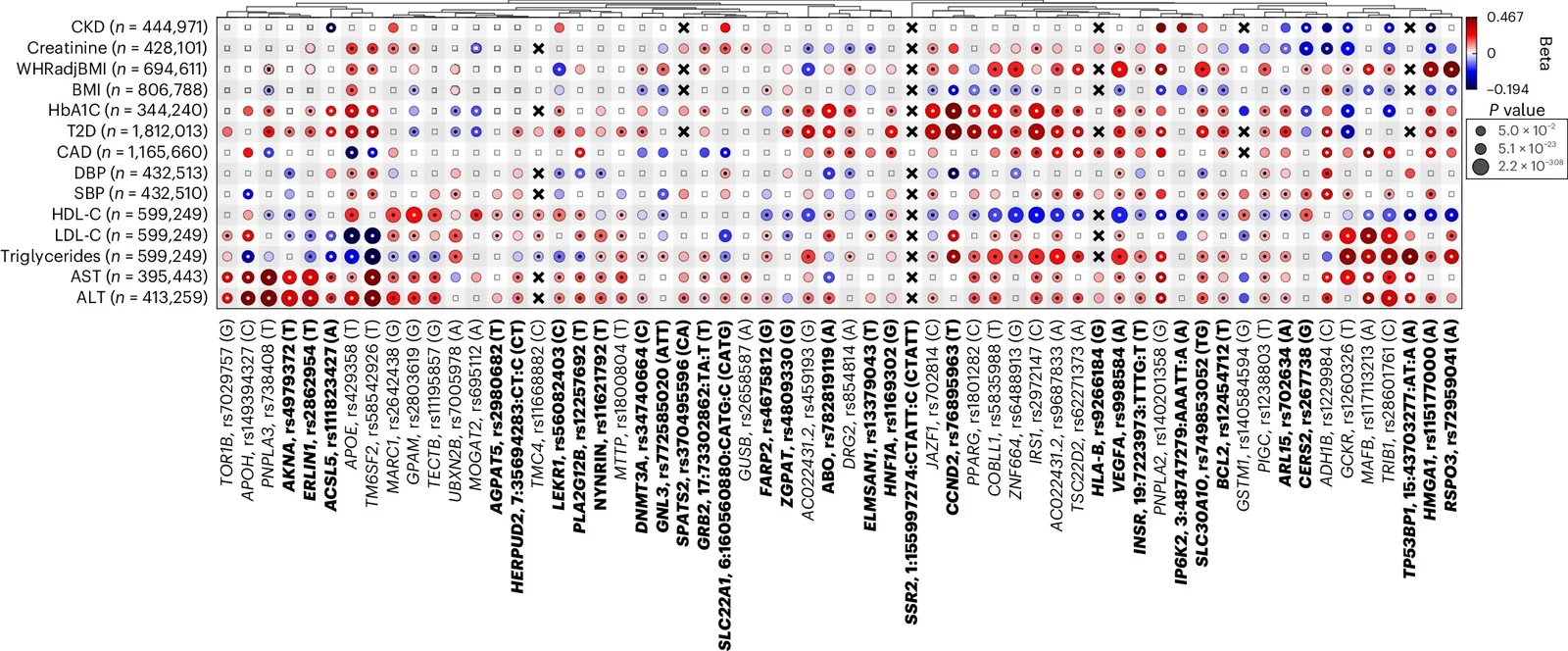
European-ancestry associations of PDFF lead variants with liver enzymes and cardiometabolic traits. Dot plot showing associations of the 60 PDFF lead variants with liver enzymes, anthropometric traits, lipids, blood pressure, glycemic traits, coronary artery disease, type 2 diabetes and chronic kidney disease in European-ancestry GWAS summary statistics. Effects are aligned to the PDFF-increasing allele. Circles indicate associations with *P* ≤ 0.05, whereas squares indicate associations with *P* > 0.05. Red indicates a positive association and blue indicates a negative association for the PDFF-increasing allele. Circle size represents the association *P* value, with larger circles indicating smaller *P* values. All *P* values are from two-sided association tests reported in the source GWASs, as described in Methods. Central marks indicate associations passing Bonferroni correction for 60 variants (*P* < 0.05/60). Exact effect estimates, standard errors and *P* values are provided in Supplementary Table 8. Crosses denote variants unavailable after exact or proxy lookup. Columns are hierarchically clustered by the cross-trait association pattern, and variant labels show the nearest gene, lead variant and PDFF-increasing allele, with newly identified loci shown in bold. CAD, coronary artery disease; CKD, chronic kidney disease; DBP, diastolic blood pressure; HDL-C, high-density lipoprotein cholesterol; LDL-C, low-density lipoprotein cholesterol; SBP, systolic blood pressure; T2D, type 2 diabetes.

We next tested the 60 independent PDFF lead variants for association with liver enzymes and cardiometabolic traits using European-ancestry GWAS summary statistics (Fig. 2 and Supplementary Table 8). Most PDFF-increasing alleles were associated with higher ALT, aspartate aminotransferase (AST), triglyceride and glycated hemoglobin (HbA1c) levels; higher waist-to-hip ratio adjusted for BMI (WHRadjBMI); type 2 diabetes; coronary artery disease and higher blood pressure; and lower high-density lipoprotein cholesterol levels. PDFF-increasing alleles were often associated with lower BMI (20/23), but higher WHRadjBMI (24/26) and type 2 diabetes risk (33/37) at Bonferroni *P* < 0.05/60, showing that the allele increasing liver triglyceride content was frequently not the BMI-increasing allele. Among the 31 newly identified variants, those replicated in our MASLD meta-analysis included several loci with previous evidence, including *CCND2* and *ARL15* for type 2 diabetes^19,20^, *CERS2* for kidney function^21^ and *BCL2* for body fat distribution^22^.

In addition, all loci had concordant effects in ancestry-specific ALT association analyses (Supplementary Note, Supplementary Table 9 and Supplementary Fig. 3).

We generated a genome-wide PRS for liver triglyceride content using SBayesRC (PRS-PDFF-GWAS), which jointly models GWAS summary statistics and functional genomic annotations to improve variant effect size estimation. This PRS was associated with higher odds of cirrhosis (odds ratio (OR) = 1.38, *P* = 1.21 × 10^−65^) and HCC (OR = 2.07, *P* = 2.52 × 10^−52^) per s.d. increase in the PRS. In addition, participants in the highest PRS decile had markedly higher odds of cirrhosis (OR = 2.71, *P* = 1.61 × 10^−29^) and HCC (OR = 7.47, *P* = 1.17 × 10^−14^) compared with those in the lowest decile. The highest decile also remained associated with higher odds when compared with the middle deciles, and associations were similar in prospective and sensitivity analyses accounting for alcohol intake and competing liver disease etiologies (Supplementary Note, Supplementary Fig. 4 and Supplementary Tables 10–12).

**Fig. 3:**
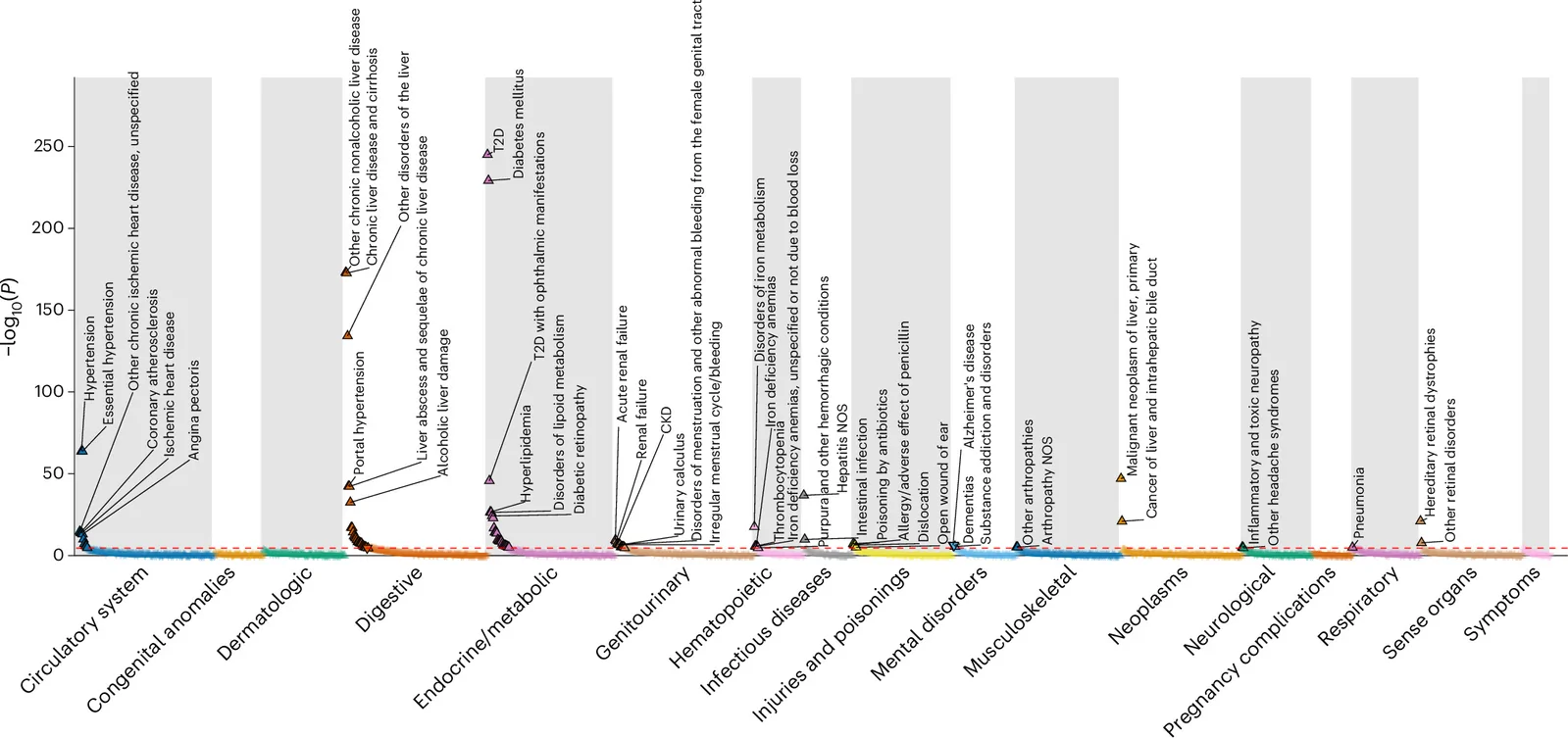
Phenome-wide association analysis of the genome-wide PRS for liver triglyceride content (PRS-PDFF-GWAS) in the UK Biobank. Associations between PRS-PDFF-GWAS and 1,579 binary phecodes were tested using logistic regression, adjusted for BMI, age, sex, age × sex, age^2^, age^2^ × sex, the first ten genomic PCs and array batch. The *x* axis shows phecodes grouped by disease category, and the *y* axis shows two-sided −log_10_(*P* value). Each point represents one phecode, and selected phecodes passing Bonferroni correction are labeled. All association analyses were performed after excluding individuals with available PDFF (*n* = 71,488). Upward-pointing triangles indicate ORs greater than 1, whereas downward-pointing triangles indicate ORs less than 1. The red dashed line indicates the Bonferroni significance threshold (*P* < 0.05/1,579). The full summary statistics are provided in Supplementary Table 13. NOS, not otherwise specified.

To test whether this PRS is associated with other diseases, we performed a phenome-wide analysis of PRS-PDFF-GWAS and identified 94 Bonferroni-significant diseases defined by phecodes (Fig. 3 and Supplementary Table 13). As expected, the PRS showed strong associations with cirrhosis and other complications of advanced liver disease and primary liver cancer, consistent with the natural history of MASLD^23^. Among extrahepatic phenotypes, the clearest pattern involved type 2 diabetes and its complications, dyslipidemia, obesity, cardiovascular disease and chronic kidney disease, consistent with the broader cardiometabolic burden of MASLD^23,24^. We also observed associations with disorders of iron metabolism and cholelithiasis, both previously linked to SLD^25,26^. The genome-wide PRS showed by far the strongest association with type 2 diabetes, followed by liver disease. We therefore repeated the phenome-wide analysis using a PRS restricted to the 60 loci identified here (PRS-PDFF). In this analysis, liver disease showed the strongest association, followed by type 2 diabetes (Supplementary Fig. 5). This contrast suggests that the genome-wide score captures additional variants with broader metabolic pleiotropic effects linking diabetes to MASLD.

To identify pathways associated with genetically predicted liver triglyceride content, we performed preranked gene set enrichment analysis of plasma proteins associated with PRS-PDFF-GWAS at a false discovery rate (FDR) below 0.05 in the UK Biobank. Reactome analysis showed enrichment for amino acid and carbohydrate metabolism, biological oxidations, sphingolipid metabolism and interleukin-10 signaling among proteins positively associated with higher genetic predisposition to liver triglyceride content. GTEx tissue enrichment showed a similar pattern, with proteins increasing with higher PRS enriched for liver expression, whereas proteins negatively associated with higher PRS were enriched for visceral adipose tissue expression (Supplementary Tables 14 and 15). We additionally performed a tissue-origin enrichment analysis using a plasma proteome distribution atlas^27^ and observed that liver-origin proteins were the top enriched tissue for both PRS-PDFF (OR = 3.03, *P*_FDR_ = 5.95 × 10^−13^) and PRS-PDFF-GWAS (OR = 2.25, *P*_FDR_ = 4.04 × 10^−7^; Supplementary Note). Inverse GTEx enrichment for visceral adipose tissue expression is consistent with recent evidence implicating reduced visceral adipocyte differentiation in MASLD^28^. The enrichment of amino acid metabolism is notable given recent evidence that amino acids can serve as a major carbon source for hepatic lipogenesis^29^. Interleukin-10 signaling was driven by pro-inflammatory cytokines and chemokines, including interleukin-6, tumor necrosis factor and CXCL10. Together, these results highlight cytokine and chemokine signaling among plasma protein changes associated with genetically predicted liver triglyceride content, consistent with their central roles in hepatic inflammation and MASLD progression^30,31^.

To further examine cell-type enrichment in the PDFF-GWAS signal, we performed Multi-marker Analysis of Genomic Annotation (MAGMA) gene-property analysis using cell-type group annotations from the Human Protein Atlas (HPA)^32^. PDFF-GWAS associations were enriched in both adipocyte- and hepatocyte-expressed genes, with stronger enrichment in adipocytes. After the fine-mapped intervals for the 60 loci were excluded, adipocyte enrichment remained significant, while hepatocyte enrichment was attenuated (Supplementary Table 17). These findings suggest that enrichment in adipocyte-expressed genes is broader in the genome-wide PDFF signal than in the 60-locus signal.

Our study identifies 60 independent loci for liver triglyceride content, including 31 not previously reported, thereby nearly doubling the number of known loci, and estimates its SNP-based heritability at 25%. Validation of 14 of the 31 previously unreported variants in clinically diagnosed MASLD indicates that MRI-derived liver triglyceride content captures inherited susceptibility relevant to clinical liver disease. The PRS, phenome-wide association and plasma protein analyses further characterized this genetic susceptibility through associations with advanced liver disease, cardiometabolic phenotypes and liver-enriched circulating proteins. Because BMI was included as a covariate, the GWAS and downstream analyses should be interpreted as genetic associations with liver triglyceride content that are not explained by generalized adiposity, although this may attenuate variants acting mainly through BMI. A striking finding is that PRS strata strongly predicted cirrhosis, whereas the corresponding increase in risk was nearly threefold greater for HCC. This is consistent with data from individuals carrying rare *APOB* variants, in whom the increase in HCC risk was greater than that for cirrhosis^33^. Taken together, these findings suggest that genetic susceptibility to hepatic lipid accumulation may contribute differently to cirrhosis and HCC risk. An important limitation is restriction to European ancestry, and larger MRI-derived PDFF studies in non-European ancestries are needed to assess the transferability of these findings. Generalizability may also be limited by the UK Biobank study because liver disease burden and major risk factors may differ across European countries^34^. The MRI-PDFF discovery analysis used UK Biobank imaging and therefore lacks replication in an independent imaging cohort. External support for the newly identified loci also varied: the MASLD meta-analysis supported 22 of the 31 loci at nominal significance, including 14 after Bonferroni correction, whereas the remaining loci should be interpreted as PDFF discovery associations until replicated in larger external imaging studies. In conclusion, this study identifies additional loci and candidate effector genes for liver triglyceride content and characterizes their associations with MASLD, cardiometabolic phenotypes, cirrhosis and HCC.

## Methods

### Ethics

This research complies with the principles outlined in the Declaration of Helsinki. The UK Biobank received ethical approval from the National Research Ethics Service Committee North West Multi-Centre Haydock (reference 16/NW/0274). Data used in this study were obtained under application number 37142. The Molecular Architecture of Fatty Liver Disease in Patients with Obesity Undergoing Bariatric Surgery (MAFALDA) study has been approved by the local research ethics committee (number 16/20). Each study participant provided written informed consent.

### UK Biobank

The UK Biobank is a large prospective study that enrolled more than 500,000 people aged 40–69 years across the UK between 2006 and 2010, providing extensive phenotypic and genetic information^18^. Ethical approval for the UK Biobank was granted by the North West Multi-Centre Research Ethics Committee (reference 16/NW/0274), and data used in this study were obtained under application number 37142. Participants of European ancestry were identified by adding participants who self-reported as Irish or any other white background, after removal of outliers based on the first six genetic principal components (PCs), to those of white British ancestry^35^. Additional exclusions were applied for participants with any of the following: more than ten putative third-degree relatives, a mismatch between self-reported and genetically determined sex, evidence of sex-chromosome aneuploidy, heterozygosity and missingness outliers, or withdrawal of consent^18^.

### Imaging protocol and analysis

We analyzed the single-slice IDEAL liver acquisition (data-field 20254) in 42,028 participants. Image handling and segmentation followed the published UK Biobank abdominal MRI pipeline from Liu et al.^8^ using the public implementations (https://archive.softwareheritage.org/browse/origin/directory/?origin_url=https://github.com/recoh/pipeline and https://archive.softwareheritage.org/browse/origin/directory/?origin_url=https://github.com/calico/ukbb-mri-sseg/). From the pipeline outputs in IDEAL space, we obtained the PDFF map in percent units and the two-dimensional liver segmentation produced by the IDEAL-specific U-Net. To increase boundary specificity, the liver mask was eroded by one voxel in two dimensions. Scans with fewer than 20 voxels inside the eroded mask were excluded. PDFF voxel values within the retained mask were constrained to the physical range 0–100%, and the participant-level PDFF was defined as the median of these values.

After PDFF extraction, we applied an additional quality-control step to remove scans with implausible PDFF maps. As a reference for setting the anomaly threshold, we used UK Biobank data-field 24352, which provides fat-referenced liver PDFF measured as the average across up to nine (minimum of three) liver regions selected to avoid inhomogeneities, major vessels and bile ducts^6^. Because this reference is average-based, we used our IDEAL mean PDFF for this calibration step, while retaining the IDEAL median PDFF as the GWAS phenotype. We tested several scan-level measures from the raw PDFF map and from the liver-mask boundary. These measures summarized the upper tail of the PDFF voxel distribution, the spread of voxel values inside the mask, the difference between the upper tail and the central PDFF value, the fraction of voxels near 0% or 100%, PDFF values immediately inside and outside the mask, in-phase signal contrast across the mask boundary, and simple mask-size and mask-location measures. These measures were chosen to flag residual artifacts after segmentation and mask erosion, including motion, boundary leakage or small clusters of spuriously high PDFF voxels^36–38^. We converted each quality-control measure to a robust *z* score using median absolute deviation scaling. We then scored scans with five unsupervised anomaly-detection methods: robust Mahalanobis distance, isolation forest, robust random cut forest, local outlier factor and one-class support vector machine. To compare methods, we converted each anomaly score to a relative rank, with higher values indicating more anomalous scans. Among participants who also had UK Biobank data-field 24352 available, provisional discordant scans were defined as scans where the absolute difference between our IDEAL mean PDFF and data-field 24352 was greater than 3.5 scaled median absolute deviations. We used fivefold cross-validation to choose the anomaly-score threshold for each candidate model, prioritizing high precision because false-positive exclusions would remove participants with genuinely high liver fat.

The final model used both the 99th percentile of the raw PDFF voxel values inside the eroded liver mask and the isolation forest method. In cross-validation, this model achieved a precision of 0.93 and flagged 0.70% of scans in the field 24352 overlap. We then applied this single threshold to the full cohort and excluded 188 scans that exceeded it from downstream analyses (Supplementary Fig. 1 and Supplementary Table 1).

### Genotypes and imputation

UK Biobank participants were genotyped on two closely related SNP arrays with >95% marker overlap. Central imputation by the UK Biobank used the Haplotype Reference Consortium, UK10K and 1000 Genomes Phase 3 reference panels. From approximately 97 million imputed variants, we retained 9,356,431 with minor allele frequency (MAF) > 1%, imputation quality (INFO) > 0.8 and Hardy–Weinberg equilibrium *P* > 1 × 10^−10^ (refs. ^18,35^).

### Genome-wide association analysis

We examined the association between 9 million imputed common variants and MRI-derived PDFF in 71,488 participants of European ancestry using nonoverlapping samples from three sources: (1) PDFF from the single-slice gradient-echo acquisition (data-field 21092; *n* = 1,200), (2) PDFF extracted from IDEAL single-slice images in the present study (*n* = 35,179) and (3) the Perspectum-derived PDFF variable (data-field 40061 (ref. ^7^); *n* = 35,109). The three GWASs were analyzed separately because the MRI acquisitions and PDFF derivation methods differed across datasets.

Participants overlapping across datasets were removed to create independent analysis groups. Those with data-field 40061 were excluded from the dataset containing PDFF extracted in the present study (*n* = 1,550), and participants present in either of these two datasets were further excluded from the gradient-echo PDFF dataset (*n* = 8,742). Association analysis was performed using a whole-genome regression model implemented in REGENIE (v4.1)^39^, adjusting for age at MRI, sex, age^2^, age × sex, age^2^ × sex, BMI at MRI, genotyping array and the first ten PCs of ancestry. Summary statistics from the three analyses were combined using fixed-effect inverse variance-weighted meta-analysis in METAL (version released on 5 May 2020) (ref. ^40^).

As a sensitivity analysis, we harmonized PDFF measures to a common scale defined by data-field 40061. Among participants with both 21092 and 40061 (*n* = 8,659), we fitted a linear regression with data-field 40061 as the outcome and used the fitted relationship to rescale PDFF from 21092. The same approach was applied to participants with both 40061 and our extracted PDFF (*n* = 1,550) to align those measurements to the 40061 scale. Association analysis was then repeated using the rescaled PDFF to verify consistency with the meta-analysis results. As an additional sensitivity analysis, associations for the PDFF lead variants were examined after further adjustment for alcohol intake at the imaging visit.

For step 1 of REGENIE, the whole-genome regression model was trained using directly genotyped common variants with MAF > 1%. Variants located within long-range LD regions or the major histocompatibility complex (MHC) region were excluded. Among the remaining variants, those with a missingness rate below 1% and Hardy–Weinberg equilibrium *P* > 1 × 10^−15^ were retained. LD pruning was then performed using a 500-kilobase (kb) window and pairwise *r*^2^ < 0.1 (ref. ^41^), leaving 146,833 markers for model fitting. PDFF values were transformed to a rank-based inverse normal distribution before analysis.

### Identifying independent variants and fine-mapping

We first defined approximately independent loci by LD clumping (PLINK v1.90b6.26; --clump-p1 5e-8 --clump-r2 0.01 --clump-kb 1000) after excluding individuals related at third degree or closer^18,41^. To detect statistically independent variants, we applied approximate stepwise model selection using the conditional and joint multiple-SNP analysis in Genome-wide Complex Trait Analysis (GCTA-COJO^42^, v1.94.0) with a 10-megabase (Mb) LD window and in-sample LD estimated from 50,000 randomly selected unrelated Europeans from the UK Biobank^35^.

Fine-mapping was carried out around the COJO lead variants (*P* < 5 × 10^−8^) by extending 1.5 Mb upstream and downstream of each lead and merging overlapping regions. We excluded the MHC region on chromosome 6 owing to its complex LD structure. We used PolyFun (v1.0.0)^9^ to estimate per-SNP heritability via an L2-regularized extension of stratified LD score regression with the baseline-LD v2.2 model (187 annotations). The resulting per-SNP priors were used in SuSiE (v0.14.2)^1^, allowing up to ten causal variants per region. LD for fine-mapping was computed from a subset of 337,000 unrelated white British UK Biobank participants.

Independent signals within each locus were taken from the fine-mapping output. The primary hit was the strongest credible set with a lead variant at *P* < 5 × 10^−8^. Secondary hits were retained only when they also reached genome-wide significance and had log Bayes factor > 2, a filter that removes additional credible sets that SuSiE can produce for a single underlying association^16^.

### Estimating heritability and genetic correlation

We quantified SNP-based heritability and evaluated confounding using LDSC (v1.0.1)^9^ with the baseline LD model version 2.2, which includes 97 functional and architecture-related annotations capturing MAF and LD structure^43^. Variants in the MHC region (chromosome 6, 25–34 Mb) were removed because of the complex LD structure in this region. We set the LDSC chisq-max parameter to 99,999 to retain variants with large effect sizes. We also estimated genetic correlation between the PDFF-GWAS meta-analysis and the clinically diagnosed MASLD GWAS meta-analysis using bivariate LDSC with European LD scores from 1000 Genomes^9^.

### *cis*-eQTL mapping and colocalization

We performed *cis*-eQTL mapping in liver (*n* = 217) bulk RNA sequencing from the MAFALDA study^1^ using tensorQTL^44^ (v1.0.9). Total RNA was extracted using the miRNeasy Advanced Mini kit (QIAGEN). Libraries were prepared and sequenced as 150-bp paired-end reads using the Illumina NovaSeq PE150 (Novogene). Read quality was assessed using FastQC (v.0.12.0, Babraham Bioinformatics), and low-quality reads and potential contaminating adapters were removed using Trimmomatic (v.0.39)^45^. Reads were aligned to the GRCh38 reference genome using STAR (v.2.7.10a)^46^, and gene-level read counts were quantified against Ensembl release 107 using RSEM (v.1.3.3)^47^. Samples with RNA integrity number < 4 or <70% uniquely mapped reads were excluded before analysis. We tested 19,381 genes that had transcripts per million > 0.1 in at least 20% of samples and raw counts > 6 in at least 20% of samples^48^. Gene counts were the trimmed mean of *M* values normalized to counts per million^49^ and inverse- normalized across the remaining samples. A total of 4,317,408 SNPs were available for the analysis based on the imputed (*R*^2^ > 0.3) and post-quality control SNPs with a MAF > 0.1 in Italians in the UK Biobank. The model included 10 genetic and 35 expression PCs to adjust for technical factors and hidden confounders. The number of expression PCs to include in the model was determined empirically on chromosome 20 to maximize the number of eGenes discovered compared with the number of PCs^50^. We used the cis_nominal mode to test all SNP–gene pairs within the 500-kb window of the start and end sites of each gene and declared significant eGenes at an FDR of 0.05.

We next conducted colocalization analysis between independent genetic loci and the *cis*-eQTL summary statistics. The coordinates of GWAS summary statistics were first converted from Build 37 to 38 using the liftOver function of the rtracklayer R package (v.1.54.0)^51^. We then tested, at each locus, whether the PDFF association signal and the *cis*-eQTL signal were consistent with a shared causal variant using COLOC-SuSiE (coloc R package^52^ v5.2.3) under a multiple-causal-variant model and default priors, restricting analyses to variants within 500 kb of the index variant. Colocalization was evaluated only for genes identified as eGenes in our *cis*-eQTL analysis, using a ±500-kb window around each eGene. If SuSiE failed to converge within 1,000 iterations, we reran colocalization using the single-causal-variant coloc model. We considered an H4 posterior probability >0.8 as strong evidence that the GWAS and *cis*-eQTL signals are driven by the same causal variant.

### Variant annotation

Independent genome-wide significant and fine-mapped variants were annotated using the Ensembl Variant Effect Predictor^53^ accessed from the REST API (https://rest.ensembl.org/).

### Candidate gene prioritization

Gene prioritization for independent genetic loci identified in the fine-mapping analysis was performed using two frameworks:

1. FLAMES^10^ (v1.1.2) integrates SNP-to-gene evidence and convergence-based evidence into a single calibrated prediction score for each fine-mapped locus. First, we ran MAGMA (v1.08)^54^ implemented in FUMA (v1.5.4)^55^, using 10,000 randomly selected European participants from the UK Biobank as the reference panel to obtain gene-level and tissue-type summary statistics. Next, we ran Polygenic Priority Score (PoPS, v0.2)^56^ using the generated MAGMA gene-level *z* scores and pathway-naive PoPS features to generate convergence-based gene-prioritization scores. We then created credible sets from our fine-mapping analysis and ran FLAMES using PoPS and MAGMA scores.
2. CALDERA^11^ combines fine-mapped GWAS variants with regulatory, expression and chromatin features in an L1-regularized logistic regression model and reports prediction probabilities for each gene at a locus. The same FLAMES inputs (except tissue-type MAGMA summary statistics) were used to run CALDERA.

Gene prioritization was complemented by colocalization with liver *cis*-eQTL summary statistics from the MAFALDA cohort. Colocalization evidence was incorporated in one of two settings: (1) as supporting evidence when FLAMES or CALDERA already prioritized the same gene or (2) as the primary source of evidence when neither FLAMES nor CALDERA prioritized a gene. We assigned a confidence score of 1 to each approach if any of the following criteria were met: FLAMES cumulative precision > 0.75, CALDERA prediction probability > 0.5 or colocalization H4 posterior probability > 0.8. The overall confidence score for each predicted effector gene was defined as the sum of these binary scores. For loci where none of these criteria were met, the top gene prioritized by either FLAMES or CALDERA was retained as a lower-confidence candidate.

### Validation

To replicate the previously unknown PDFF loci identified in this study, we tested their associations with clinically diagnosed (International Classification of Diseases (ICD)-9 or ICD-10 codes) MASLD, cirrhosis and HCC in European-ancestry participants. For MASLD and HCC, we first gathered GWAS summary statistics from the MVP, FinnGen Release 12, deCODE and Intermountain^15–17^. In the UK Biobank, to match the genome build (GRCh38) of the summary statistics from these four studies, we used the UK Biobank release imputed to the Trans-Omics for Precision Medicine reference panel, which is based on deep-coverage whole-genome sequences from the National Heart, Lung, and Blood Institute Trans-Omics for Precision Medicine program^57^. Only variants with MAF > 1% and Minimac *R*^2^ > 0.65 were retained for the association analyses. To avoid sample overlap, we excluded European-ancestry individuals with an available PDFF measurement (*n* = 71,488). Association analysis was performed using a whole-genome regression model implemented in REGENIE (v4.1)^39^, adjusting for age, sex, age^2^, age × sex, age^2^ × sex, genotyping array and the first ten PCs of ancestry. Summary statistics from the UK Biobank, MVP, FinnGen, deCODE and Intermountain were then combined using fixed-effect inverse variance-weighted meta-analysis in METAL (version released on 5 May 2020)^40^. We retained only variants that were present in at least three studies. The description of each study and the definitions of MASLD and HCC are provided in Supplementary Table 7.

For cirrhosis, we used GWAS meta-analysis summary statistics from Ghouse et al.^58^ in individuals of European ancestry from 9 studies (15,225 cases and 1,564,786 controls).

For PDFF lead variants that were not available in the MASLD, HCC or cirrhosis GWAS meta-analysis summary statistics, we selected a proxy variant. Proxy selection was performed using LDproxy^59^, choosing variants in LD (*r*^2^ > 0.4) with the PDFF lead variant in Europeans from the 1000 Genomes Project. If no such proxy was found by LDproxy, we searched within the UK Biobank using a 250-kb window around the lead variant in a subset of 337,000 unrelated white British UK Biobank participants. When multiple proxies were available, we selected the variant with the highest LD and most relevant functional consequence.

### Association of PDFF lead variants with liver enzymes and cardiometabolic traits

Associations of the 60 independent PDFF lead variants with liver enzymes and cardiometabolic traits were examined using GWAS summary statistics from the MVP for ALT, AST, HbA1c, creatinine, systolic blood pressure and diastolic blood pressure^17^; the GIANT/UK Biobank anthropometric GWAS meta-analysis for BMI and WHRadjBMI^60^; the GWAS meta-analysis of the UK Biobank and Estonian Biobank for triglycerides, high-density lipoprotein cholesterol and low-density lipoprotein cholesterol^61^; Aragam et al.^62^ for coronary artery disease; Suzuki et al.^63^ for type 2 diabetes; and Wuttke et al.^21^ for chronic kidney disease. ALT associations were also examined in multi-ancestry summary statistics from the MVP and BioBank Japan.

For PDFF lead variants that were not available in a target GWAS summary statistic, we selected a proxy variant. In addition, for European-ancestry summary statistics, proxy selection was also performed for exact matches when the difference between the effect-allele frequency in the PDFF-GWAS and the target summary statistic was above 0.3. Proxy selection for Europeans was first performed using LD estimated in 337,000 unrelated European UK Biobank participants within 250 kb of the PDFF lead variant. If no proxy was identified, or for non-European ancestry summary statistics, proxy selection was performed using LDproxy with the corresponding ancestry from the 1000 Genomes Project. Proxies with *r*^2^ > 0.8 were retained. When multiple proxies were available, the variant with the highest LD and most relevant functional consequence was selected.

### PRS construction

We generated two PRSs for liver triglyceride content. PRS-PDFF was calculated as the weighted sum of genotype dosages across the 60 independent lead variants identified in the meta-analysis, using the corresponding effect estimates as weights. We also generated a genome-wide score, PRS-PDFF-GWAS, using SBayesRC, which integrates GWAS summary statistics with functional genomic annotations to estimate joint SNP effects^64^. SBayesRC was run in Genome-Wide Complex Trait Bayesian analysis (v2.5.2) using an LD reference panel of 20,000 unrelated UK Biobank individuals of European ancestry and BaselineLD v2.2 annotations provided by the authors. The resulting joint SNP effect estimates were then used as genome-wide weights in PLINK (v2.0)^41^ to calculate the score from imputed genotype dosages.

### Association of PRS-PDFF-GWAS with end-stage liver disease

We tested the association of PRS-PDFF-GWAS with cirrhosis and HCC in the UK Biobank. Cirrhosis was defined using ICD-10 codes K70.3, K70.4, K72.1, K72.9, K74.1, K74.2, K74.6, K76.6, K76.7, I85.0 and I85.9, and HCC was defined using ICD-10 code C22.0, based on hospitalization records (data-field 41270) and underlying primary and secondary causes of death (data-fields 40001 and 40002). Individuals with chronic viral hepatitis diagnoses (ICD-10 codes B18.0, B18.1, B18.2, B18.8, B18.9, B19.0 and B19.9) were excluded from both analyses (*n* = 450). Association analyses were performed after excluding individuals with available PDFF measurements (*n* = 71,488). We tested PRS-PDFF-GWAS on the standardized scale and by modeling PRS deciles as categorical predictors, using either decile 1 or the middle deciles (deciles 5 and 6) as the reference group. Case–control analyses were performed using logistic regression, and prospective analyses were performed using Cox proportional hazards regression with age as the underlying timescale. Associations are reported as ORs or hazard ratios with 95% confidence intervals. All models were adjusted for age, sex, age^2^, age × sex, age^2^ × sex, BMI, the first ten genomic PCs and array batch. For prospective analyses, individuals with the relevant diagnosis at baseline were excluded before analysis. Median attained age at event or censoring, estimated using the reverse Kaplan–Meier method, was 70.8 years. The proportional hazards assumption was assessed using Schoenfeld residuals and showed no evidence of violation.

### Phenome-wide association analysis

We performed phenome-wide association analyses of PRS-PDFF and PRS-PDFF-GWAS in the UK Biobank. Phecodes were defined using the UKB Phecode v1.2b1 ICD mapping from Pan-UKB^65^, restricted to ICD-10 codes. Individuals carrying the mapped ICD-10 codes in hospital inpatient or death registry records were defined as cases, and when exclusion phecodes were specified, the corresponding ICD-10 codes were used to exclude individuals from the control group. For sex-specific phecodes, individuals of the opposite sex were also excluded. After removing the phenotypes with fewer than 10 cases, 1,579 binary phecodes were included for association analyses. We tested the association of PRS-PDFF and PRS-PDFF-GWAS with each phecode after excluding individuals with an available PDFF measurement (*n* = 71,488). Each PRS was standardized before analysis. Associations were tested using logistic regression adjusted for age, sex, age^2^, age × sex, age^2^ × sex, BMI, genotyping array and the first ten PCs of ancestry. For sex-specific phecodes, sex and its interaction terms with age were omitted from the model. Statistical significance was defined as Bonferroni-corrected *P* < 0.05/1,579 (3.17 × 10^−5^) for each PRS.

### Plasma proteomic analysis

We used plasma proteomic data from the UK Biobank Pharma Proteomics Project, in which 2,923 unique proteins were measured using the Olink Explore 3072 platform^66^. Association analyses were performed after excluding individuals with an available PDFF measurement (*n* = 71,488). We tested the association between PRS-PDFF-GWAS and rank-based inverse normal transformed plasma protein levels by linear regression, adjusting for age, sex, age^2^, age × sex, age^2^ × sex, BMI, Olink batch, genotyping array and the first ten genetic PCs^67^. *P* values were corrected across proteins using the Benjamini–Hochberg FDR. Preranked gene set enrichment analysis was then performed on the proteins that passed FDR correction, ranking proteins by the beta coefficients from the proteome association analysis. Enrichment analysis was performed against the Reactome Pathways and GTEx Tissues V8 gene-set collections using GSEApy (v1.0.5)^68^.

Moreover, we examined whether PRS-associated plasma proteins were enriched for tissue-origin annotations. Tissue-origin annotations were obtained from a recent plasma proteome atlas that inferred the likely tissue sources of circulating proteins across 18 vascularized organs and 8 major blood cell types^27^. To assess whether the tissue-origin patterns observed for the PDFF PRS were specific to liver triglyceride genetic susceptibility, we tested the associations of plasma proteins with PRS for atrial fibrillation, BMI, coronary artery disease, cardiovascular disease, glycated hemoglobin, high-density lipoprotein cholesterol, hypertension, low-density lipoprotein cholesterol, circulating triglycerides, type 1 diabetes and type 2 diabetes^69^. We mapped the tissue-origin labels to the Olink proteins included in the PRS–protein association analyses. Proteins annotated as common or without a tissue label were excluded from the enrichment analyses. PRS-associated proteins were defined as those with a Benjamini–Hochberg FDR-adjusted *P* < 0.05. For each tissue, enrichment was tested using a one-sided Fisher exact test comparing tissue-assigned and nontissue-assigned proteins among PRS-associated and nonassociated proteins.

### MAGMA gene-property analysis

To examine whether PDFF genetic associations were enriched in genes expressed in specific cell types, we performed MAGMA (v1.10)^54^ gene-property analysis using single-cell expression annotations from the HPA^32^. The HPA single-cell resource integrates single-cell and single-nucleus RNA sequencing data from healthy human tissues and provides expression matrices for cell types and broader cell-type groups. We used the HPA cell-type group annotations for this analysis. MAGMA gene-level statistics were generated from the PDFF-GWAS meta-analysis summary statistics using GRCh37 gene coordinates and the European 1000 Genomes reference panel. The MHC region on chromosome 6 (25–34 Mb) was excluded from all analyses. We generated three MAGMA inputs: the full GWAS, the GWAS after excluding the major locus intervals and the major locus intervals only. The major locus intervals were the SuSiE fine-mapping regions. For each HPA annotation, normalized counts per million (nCPM) were mapped to the MAGMA gene identifiers and transformed as log_2_(nCPM + 1). When multiple HPA entries mapped to the same MAGMA gene and annotation, the maximum nCPM value was retained. Genes without mapped HPA expression were removed from the gene-property models. For each HPA cell-type group, we tested whether genes with higher expression in that annotation had stronger PDFF gene-level association using a one-sided positive MAGMA gene-property model. MAGMA models included the default internal gene-level covariates for gene size, gene density, inverse mean minor allele count and their log-scaled terms. To reduce enrichment driven by generally higher expression across annotations, we additionally included the average expression across HPA cell-type groups as a hidden conditioning covariate. *P* values were corrected using the Benjamini–Hochberg FDR separately across the 53 HPA cell-type groups tested in each MAGMA analysis.

### Reporting summary

Further information on research design is available in the Nature Portfolio Reporting Summary linked to this article.

## Online content

Any methods, additional references, Nature Portfolio reporting summaries, source data, extended data, supplementary information, acknowledgements, peer review information; details of author contributions and competing interests; and statements of data and code availability are available at https://doi.org/10.1038/s41588-026-02794-6.

## Supplementary information

Supplementary Information Supplementary Figs. 1–5 and Supplementary Note.

Reporting Summary

Peer Review File

Supplementary Tables Supplementary Tables 1–17.

## Data Availability

All data associated with this study are present in the paper or the Supplementary Information. The PDFF meta-analysis summary statistics have been deposited in the National Human Genome Research Institute–European Bioinformatics Institute GWAS Catalog under accession GCST90994130. For the UK Biobank, all individual-level phenotype and genotype data are available via a formal application to the UK Biobank at http://www.ukbiobank.ac.uk. The ethical approval of the MAFALDA study restricts the public sharing of individual data. However, researchers may submit a proposal to access liver biopsy data from the MAFALDA cohort, either in raw or analyzed form, between 9 months and 36 months after publication. Proposals should be directed to S.R., who will review each request to assess data availability. Responses will be provided within 8 weeks of receiving the request. It is important to note that patient-related data may be restricted owing to confidentiality regulations. If approved for sharing, data will be transferred under a material transfer agreement. The following online databases have been used: GWAS Catalog (https://www.ebi.ac.uk/gwas/) and baseline LD model (https://data.broadinstitute.org/alkesgroup/LDSCORE/). Summary statistics for MVP are available under the dbGaP study accession phs002453.v1.p1 at https://ftp.ncbi.nlm.nih.gov/dbgap/studies/phs002453/analyses/HARE/. FinnGen GWAS summary statistics are publicly available following registration at https://www.finngen.fi/en/access_results. Summary statistics for deCODE and Intermountain are publicly available following registration at https://www.decode.com/summarydata/.

## Code availability

All code and scripts used for the analyses are available via GitHub at https://github.com/Ojami/PDFF-META-GWAS and Zenodo at https://doi.org/10.5281/zenodo.22672918 (ref. ^70^). Analyses were performed using the MATLAB Genetics Toolbox (v1.0.0; https://github.com/Ojami/MAGE). MATLAB R2024b was used under an academic license. Publicly available tools used in this study are as follows: METAL (version released on 5 May 2020), https://genome.sph.umich.edu/wiki/METAL; REGENIE v4.1, https://github.com/rgcgithub/regenie; PLINK v1.90b6.26, https://www.cog-genomics.org/plink/; GCTA-COJO v1.94.0, https://yanglab.westlake.edu.cn/software/gcta/#COJO; LDSC v1.0.1, https://github.com/bulik/ldsc/; PolyFun v1.0.0, https://github.com/omerwe/polyfun; SuSiE v0.14.2, https://github.com/stephenslab/susieR; rtracklayer v1.54.0, https://bioconductor.org/packages/release/bioc/html/rtracklayer.html; coloc v5.2.3, https://github.com/chr1swallace/coloc; Ensembl Variant Effect Predictor REST API, https://rest.ensembl.org/; FUMA v1.5.4 and MAGMA v1.08, https://fuma.ctglab.nl/; LDproxy, https://ldlink.nih.gov/?tab=ldproxy; R v4.0.2, https://www.r-project.org/; FLAMES v1.1.2, https://github.com/Marijn-Schipper/FLAMES; CALDERA, https://github.com/kheilbron/caldera; PoPS v0.2, https://github.com/FinucaneLab/pops; tensorQTL v1.0.9, https://github.com/broadinstitute/tensorqtl; FastQC v0.12.0, https://www.bioinformatics.babraham.ac.uk/projects/fastqc/; Trimmomatic v0.39, http://www.usadellab.org/cms/?page=trimmomatic; STAR v2.7.10a, https://github.com/alexdobin/STAR; and RSEM v1.3.3, https://github.com/deweylab/RSEM.
