# Supplementary material for: Genome-wide analyses identify 60 loci associated with liver triglyceride content
Source: Nat Genet. 2026 Oct 21;58(11):2772–7. doi: 10.1038/s41588-026-02794-6 (PMC13754591; doi:10.1038/s41588-026-02794-6)
Supplement: Supplementary file 1 [file 41588_2026_2794_MOESM1_ESM.pdf]

nature genetics

Supplementary information

https://doi.org/10.1038/s41588-026-02794-6

Genome-wide analyses identify 60 loci associated with liver triglyceride content

In the format provided by the authors and unedited

Supplementary Note

Prior evidence for the PDFF loci

When we compared these findings with our recent multi-adiposity adjusted GWAS of PDFF[1] including 36,394 participants, 31 of the 60 independent associations were not previously reported (Supplementary Table 2). Some of these loci had been reported previously in studies of MASLD or chronic ALT. *ERLIN1* was reported in a study combining PDFF and MASLD, although its association with PDFF alone was not genome-wide significant[2]. *INSR* was reported in a MASLD meta-analysis combining imaging and clinically defined phenotypes[3]. In addition, the *SLC30A10*, *AKNA*, *ERLIN1*, *HNF1A* and *NYNRIN* loci were previously associated with chronic ALT in Europeans[4].

Additional adjustment for alcohol intake

After additional adjustment for alcohol intake, effect estimates were highly similar to the primary GWAS (Pearson $r = 0.999$), and all 60 lead variants had directions of effect concordant with the primary GWAS. Fifty-eight variants remained genome-wide significant, whereas the SSR2 ($P = 1.3 \times 10^{-7}$) and ARL15 ($P = 7.5 \times 10^{-8}$) loci were just above the genome-wide significance threshold (Supplementary Table 4).

Associations with cirrhosis and hepatocellular carcinoma

We examined the association of variants not previously reported with cirrhosis and hepatocellular carcinoma (HCC). For HCC, we meta-analyzed summary statistics from the same studies used for MASLD (4,840 cases and 1,618,568 controls; Supplementary Table 6B). For cirrhosis, we used summary statistics from the GWAS meta-analysis by Ghouse et al.[5] in individuals of European ancestry (15,225 cases and 1,564,786 controls). The variants at the *GRB2* and *ERLIN1* loci were associated with cirrhosis and HCC, respectively, after correction for multiple testing. In addition, 8 variants showed nominal associations with cirrhosis and 7 with HCC, all with concordant directions of effect as compared to PDFF (Supplementary Table 6C).

Ancestry-specific ALT associations

We further examined ALT associations in ancestry-specific MVP summary statistics and BioBank Japan (Supplementary Table 9 and Supplementary Fig. 3). Forty-two loci were associated with ALT in Europeans ($P < 0.05/60$), all concordant with the PDFF discovery effect. In African and admixed American ancestries, the *AKNA*, *ERLIN1* and *COBLL1* loci were associated with ALT at $P < 0.05/60$, as were the *GPAM* and *TRIB1* loci in admixed American and Japanese ancestries. Overall, 37 loci showed nominal ALT associations and 16 were associated at $P < 0.05/60$ in non-European ancestries, all with concordant directions.

Prospective and sensitivity analyses of the polygenic risk score

We generated a genome-wide polygenic risk score (PRS) for liver triglyceride content using SBayesRC (PRS-PDFF-GWAS), which jointly models GWAS summary statistics and functional genomic annotations to improve variant effect size estimation. This PRS was associated with higher odds of cirrhosis (OR=1.38, P=1.21E-65) and hepatocellular carcinoma (OR=2.07, P=2.52E-52) per s.d. increase in the PRS. In addition, participants in the highest PRS decile had markedly higher odds of cirrhosis (OR = 2.71, P= 1.61E-29) and hepatocellular carcinoma (OR = 7.47, P= 1.17E-14) compared to those in the lowest decile. Additionally, when compared with the middle deciles, the highest decile remained associated with higher odds of cirrhosis (OR = 2.01, P=1.22E-27) and hepatocellular carcinoma risk (OR = 3.80, P=9.52E-19). In prospective analyses, the PRS showed a similar risk of developing cirrhosis and hepatocellular carcinoma as observed in the case-control analyses (Supplementary Fig. 4; Supplementary Tables 10 and 11). Similar associations were observed in sensitivity analyses with additional adjustment for alcohol intake, exclusion of individuals above the alcohol intake thresholds for metabolic dysfunction and alcohol-associated liver disease or alcohol-related liver disease, or additional endpoint exclusions for competing liver disease etiologies (Supplementary Table 12).

Tissue-origin enrichment analysis

We additionally performed a tissue-origin enrichment analysis using a plasma proteome distribution atlas[6] and observed that liver-origin proteins were the top enriched tissue for both PRS-PDFF (OR = 3.03, $P_{FDR} = 5.95 \times 10^{-13}$) and PRS-PDFF-GWAS (OR = 2.25, $P_{FDR} = 4.04 \times 10^{-7}$). To assess whether this liver signal was specific to PDFF genetic susceptibility, we performed the same enrichment analysis for relevant cardiometabolic PRSs[7] (see Methods). PRS-PDFF showed the strongest liver-origin enrichment among the PRSs tested, whereas liver enrichment for PRS-PDFF-GWAS was similar to that observed for CAD PRS (Supplementary Table 16).

Supplementary References

1. Jamialahmadi, O. *et al.* Partitioned polygenic risk scores identify distinct types of metabolic dysfunction-associated steatotic liver disease. *Nat Med* **30**, 3614-3623 (2024).
2. Sveinbjornsson, G. *et al.* Multiomics study of nonalcoholic fatty liver disease. *Nat Genet* **54**, 1652-1663 (2022).
3. Chen, Y. *et al.* Genome-wide association meta-analysis identifies 17 loci associated with nonalcoholic fatty liver disease. *Nat Genet* (2023).
4. Vujkovic, M. *et al.* A multiancestry genome-wide association study of unexplained chronic ALT elevation as a proxy for nonalcoholic fatty liver disease with histological and radiological validation. *Nat Genet* **54**, 761-771 (2022).
5. Ghouse, J. *et al.* Integrative common and rare variant analyses provide insights into the genetic architecture of liver cirrhosis. *Nat Genet* **56**, 827-837 (2024).
6. Malmström, E. *et al.* Human proteome distribution atlas for tissue-specific plasma proteome dynamics. *Cell* **188**, 2810-2822.e16 (2025).
7. Thompson, D.J. *et al.* A systematic evaluation of the performance and properties of the UK Biobank Polygenic Risk Score (PRS) Release. *PLoS One* **19**, e0307270 (2024).

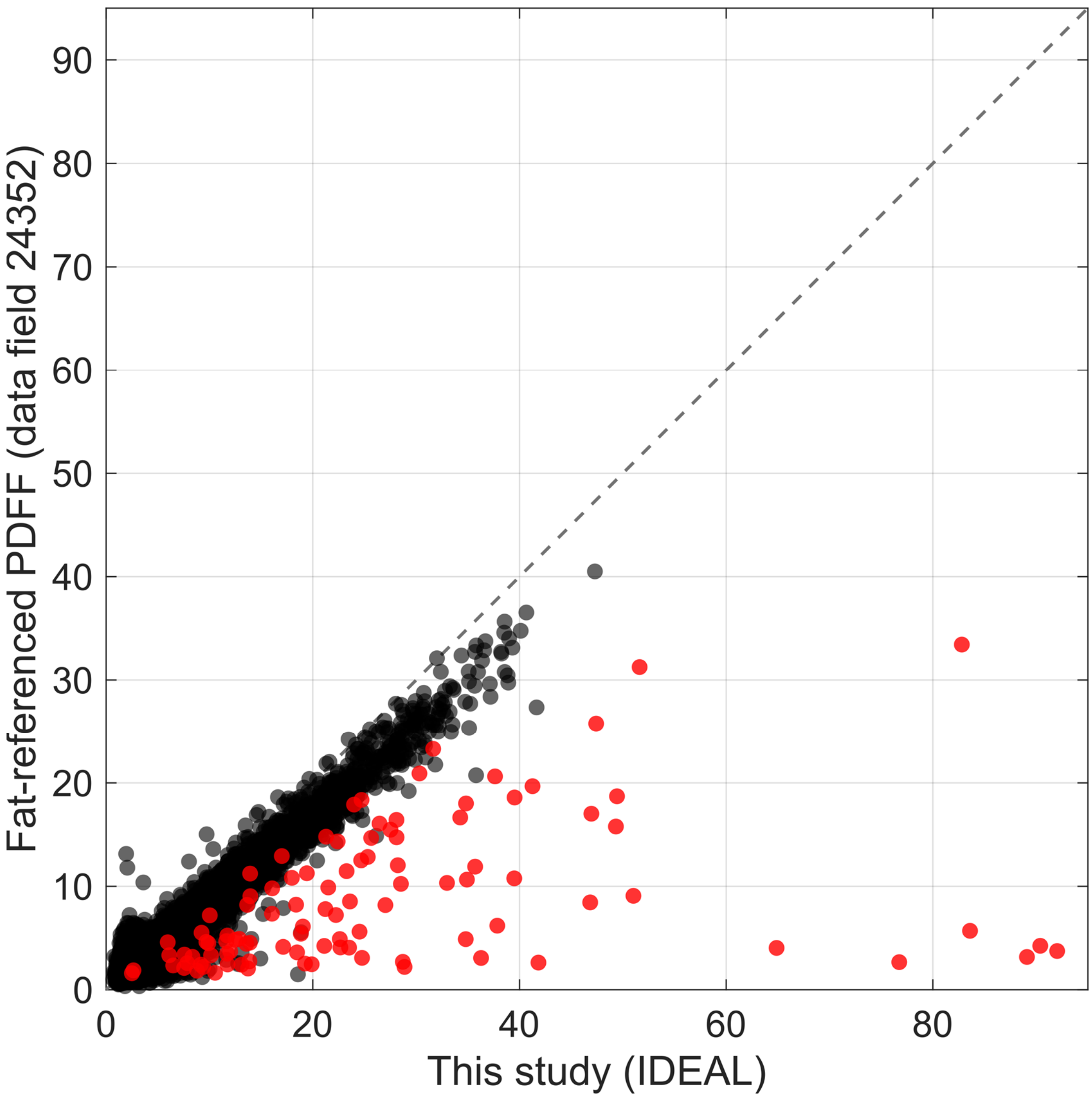

Supplementary Fig. 1. Agreement between IDEAL-derived PDFF and UK Biobank data-field 24352 after quality-control filtering. Scatter plot comparing IDEAL mean PDFF with UK Biobank data-field 24352 among participants with both measurements available (n = 15,236). The plotted comparison uses IDEAL mean PDFF because data-field 24352 is an average-based liver PDFF measure. The dashed line shows the identity line. Black points show scans retained after quality control (n = 15,135), and red points show scans excluded by the final anomaly-detection filter within this overlap (n = 101). In the full IDEAL-derived PDFF dataset, the final filter excluded 188 scans. Among retained scans, IDEAL mean PDFF correlated with data-field 24352 at R = 0.971, with MAE = 1.13% and 95th percentile absolute difference = 3.36%. The IDEAL median PDFF used as the GWAS phenotype showed similar agreement with data-field 24352 among retained scans (R = 0.980, MAE = 0.99%, 95th percentile absolute difference = 3.30%). Abbreviations: MAE, mean absolute error; PDFF, proton density fat fraction; R, Pearson correlation coefficient.

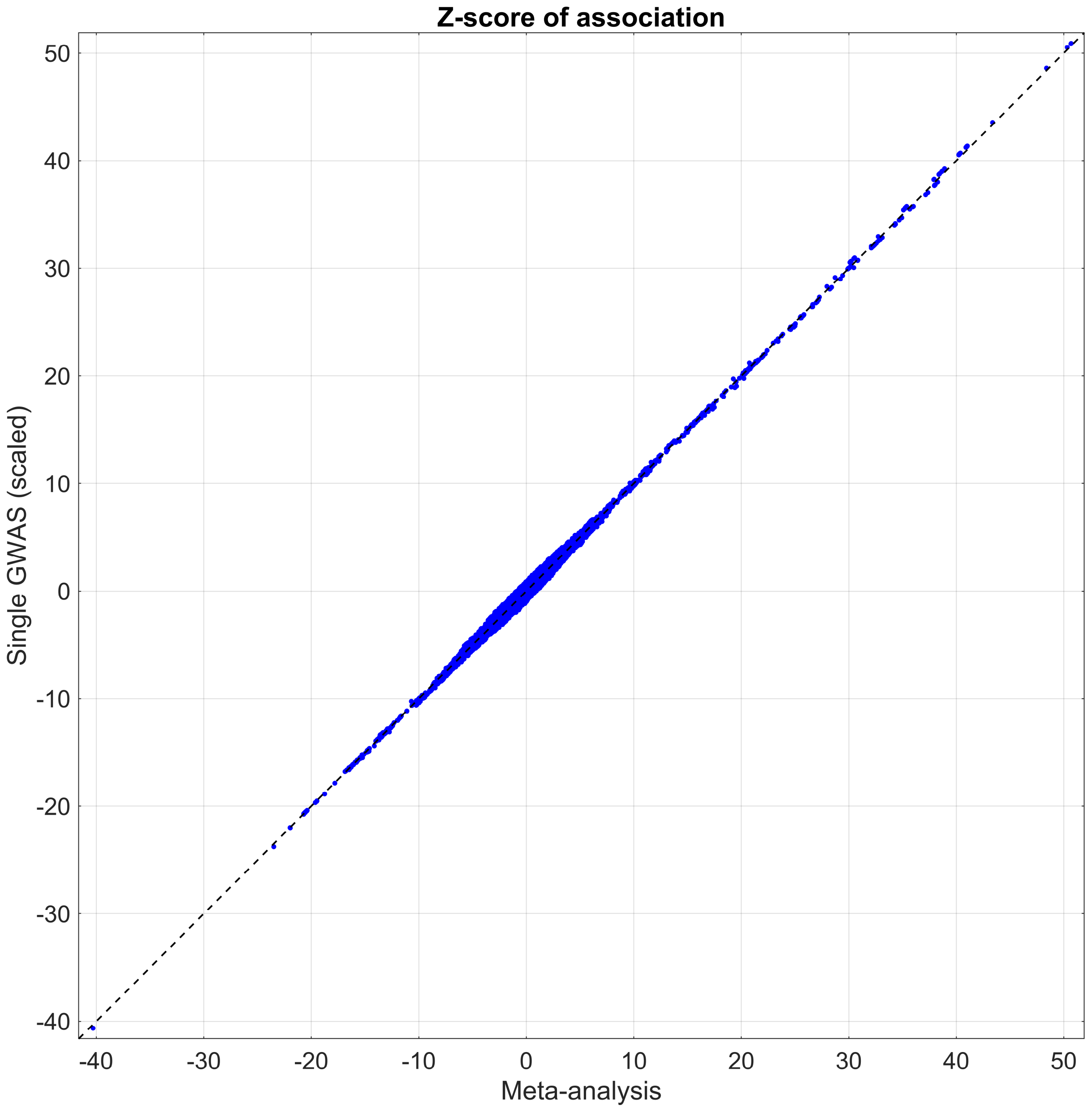

Supplementary Fig. 2. Concordance between GWAS results using original and harmonized PDFF measures. Scatter plot showing Z scores of association from the primary meta-analysis of PDFF across the three imaging-derived datasets (x-axis) versus Z scores from the sensitivity analysis using PDFF measures harmonized to a common scale defined by UK Biobank data-field 40061 (y-axis). Both meta-analyses were performed in the same participants (n = 71,488). PDFF values from data-field 21092 and the extracted PDFF measure were rescaled to the data-field 40061 scale using linear regression in participants with overlapping measurements. The dashed line indicates the line of identity.

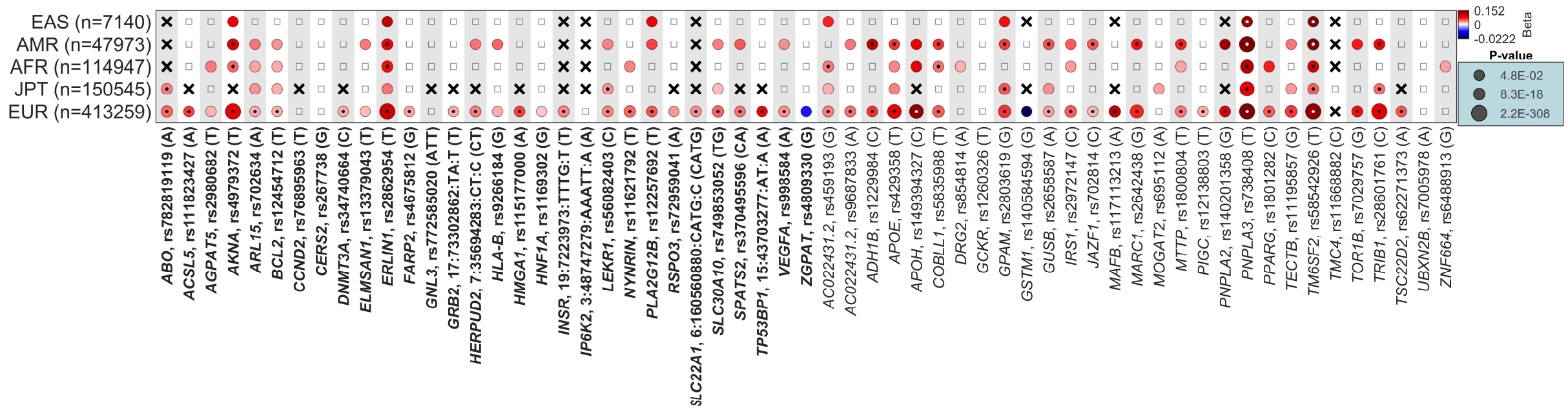

Supplementary Fig. 3. Multi-ancestry association of PDFF lead variants with alanine aminotransferase. Dot plot showing associations of the 60 PDFF lead variants with ALT in European, African, admixed American and East Asian participants from MVP and Japanese participants from BioBank Japan. Effects are aligned to the PDFF-increasing allele. Red indicates higher ALT and blue indicates lower ALT for the PDFF-increasing allele. Circles indicate associations with P ≤ 0.05, whereas squares indicate associations with P > 0.05. Circle size represents the association P value, with larger circles indicating smaller P values. Central marks indicate associations at P < 0.05/60. Crosses denote variants unavailable in the corresponding ancestry after exact or proxy lookup. Variant labels show the nearest gene, lead variant and PDFF-increasing allele, with newly identified loci shown in bold. Effect estimates, standard errors, exact P values and sample sizes are provided in Supplementary Table 9.

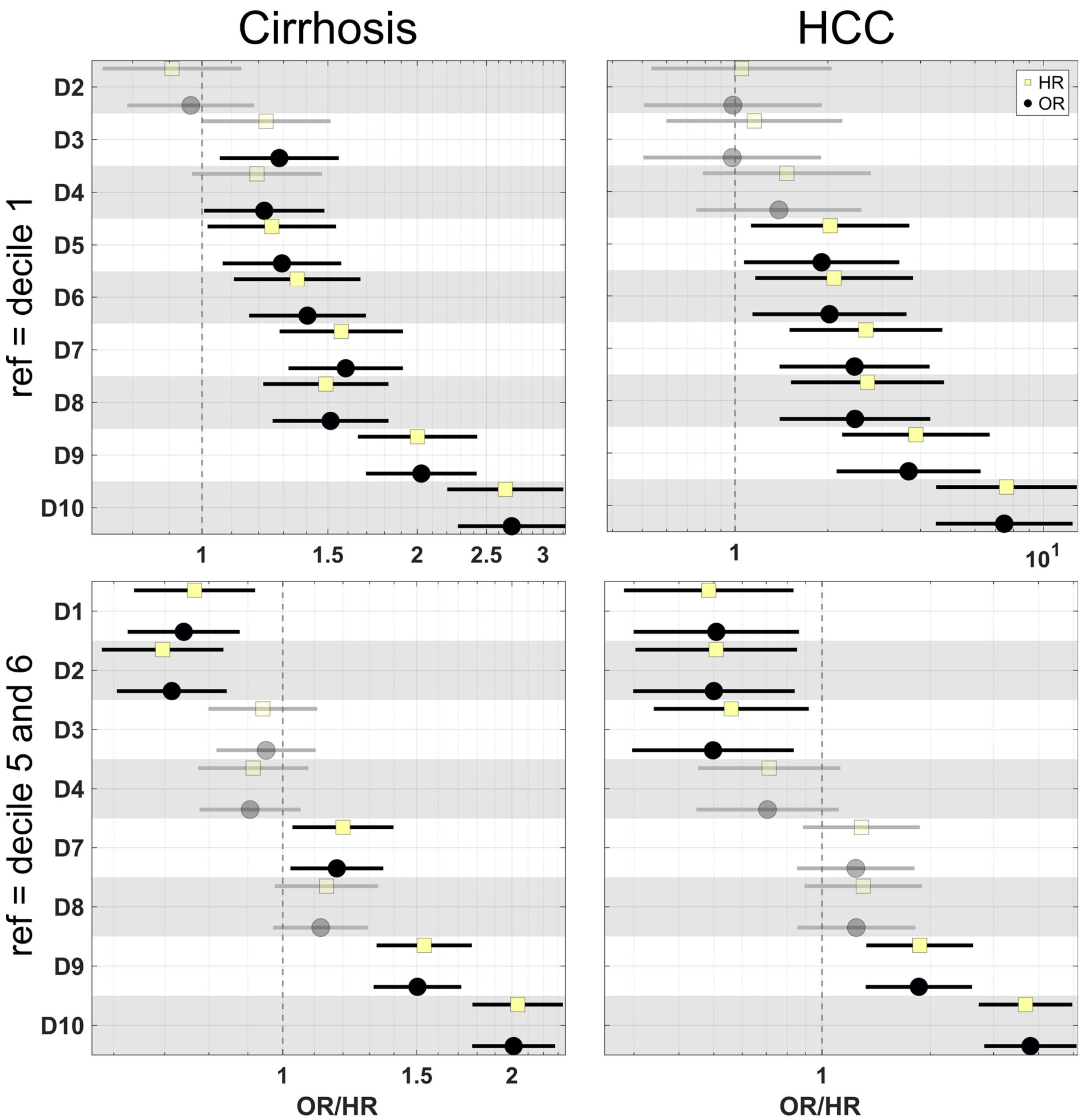

Supplementary Fig. 4. Associations of PRS-PDFF-GWAS deciles with end-stage liver disease in the UK Biobank. Associations between deciles of the genome-wide PRS for liver fat content (PDFF) and cirrhosis or hepatocellular carcinoma (HCC) were tested using logistic (OR, odds ratio) or Cox proportional hazards (HR, hazard ratio) regression models. Deciles were modeled using either decile 1 or the combined deciles 5 and 6 as the reference group. Effect estimates are shown as ORs or HRs, with 95% confidence intervals. Case-control analyses are shown as ORs and prospective analyses as HRs. All analyses were adjusted for BMI, age, sex, age x sex, age², age² x sex, the first 10 genomic principal components, and array batch. Individuals with available PDFF were excluded from analyses. The dashed vertical line indicates OR/HR = 1. Exact sample sizes and full association statistics are provided in Supplementary Table 10. Opaque symbols and black confidence intervals indicate P ≤ 0.05, whereas faded symbols and gray confidence intervals indicate P > 0.05.

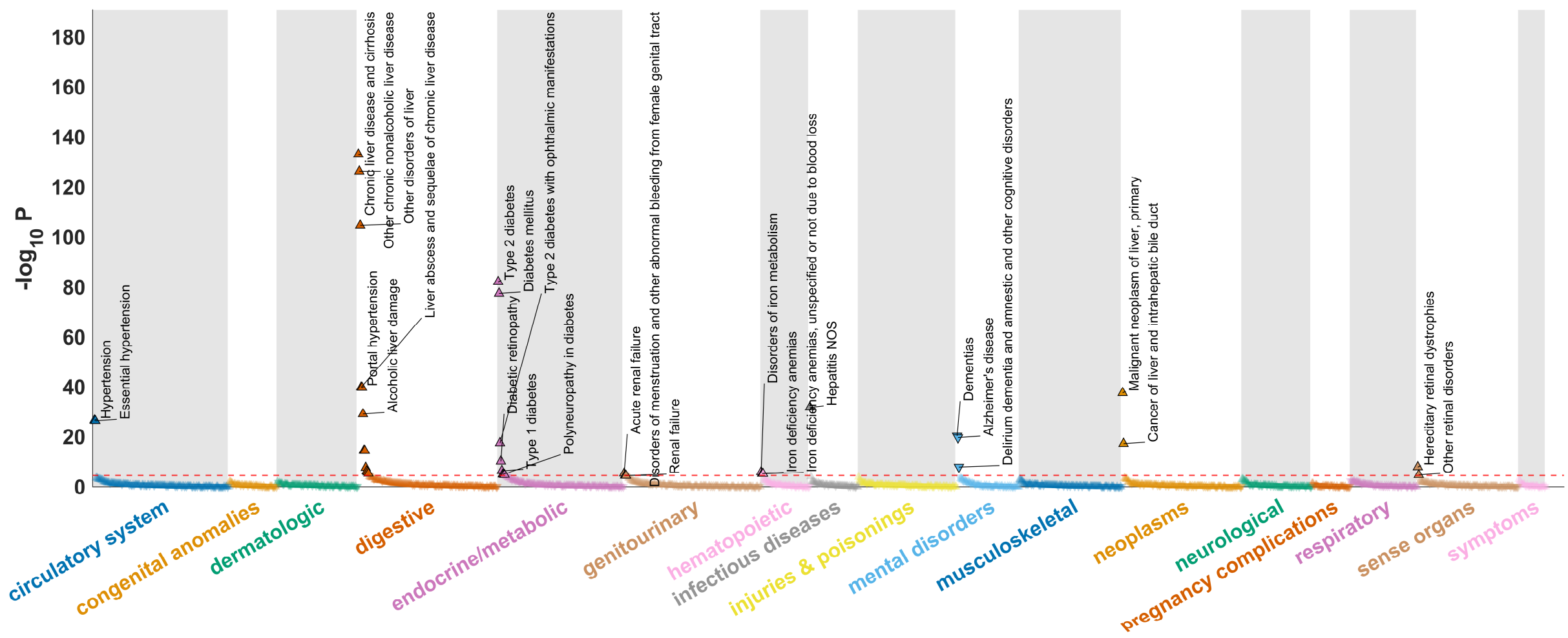

Supplementary Fig. 5. Phenome-wide association analysis of the polygenic risk score for liver triglyceride content (PRS-PDFF) in the UK Biobank. Associations between PRS-PDFF and 1,579 binary phecodes were tested using logistic regression, adjusted for BMI, age, sex, age×sex, age², age²×sex, the first 10 genomic principal components, and array batch. The x-axis shows phecodes grouped by disease category and the y-axis shows two-sided -log10 P value. Each point represents one phecode, and selected phecodes passing Bonferroni correction are labeled. All association analyses were performed after excluding individuals with available PDFF (n = 71,488). Upward-pointing triangles indicate odds ratios greater than 1, whereas downward-pointing triangles indicate odds ratios less than 1. The red dashed line indicates the Bonferroni significance threshold (P < 0.05/1,579). Full summary statistics are provided in Supplementary Table 13.
