# Supplementary material for: Genome-wide analyses identify 60 loci associated with liver triglyceride content
Source: Nat Genet. 2026 Oct 21;58(11):2772–7. doi: 10.1038/s41588-026-02794-6 (PMC13754591; doi:10.1038/s41588-026-02794-6)
Supplement: Supplementary file 2 [file 41588_2026_2794_MOESM2_ESM.pdf]

nature portfolio

Corresponding author(s): Oveis Jamialahmadi
Stefano Romeo

Last updated by author(s): Sep 10, 2026

Reporting Summary

Nature Portfolio wishes to improve the reproducibility of the work that we publish. This form provides structure for consistency and transparency in reporting. For further information on Nature Portfolio policies, see our Editorial Policies and the Editorial Policy Checklist.

Statistics

For all statistical analyses, confirm that the following items are present in the figure legend, table legend, main text, or Methods section.

n/a	Confirmed	
☐	☒	The exact sample size (*n*) for each experimental group/condition, given as a discrete number and unit of measurement
☒	☐	A statement on whether measurements were taken from distinct samples or whether the same sample was measured repeatedly
☐	☒	The statistical test(s) used AND whether they are one- or two-sided<br>*Only common tests should be described solely by name; describe more complex techniques in the Methods section.*
☐	☒	A description of all covariates tested
☐	☒	A description of any assumptions or corrections, such as tests of normality and adjustment for multiple comparisons
☐	☒	A full description of the statistical parameters including central tendency (e.g. means) or other basic estimates (e.g. regression coefficient) AND variation (e.g. standard deviation) or associated estimates of uncertainty (e.g. confidence intervals)
☐	☒	For null hypothesis testing, the test statistic (e.g. *F*, *t*, *r*) with confidence intervals, effect sizes, degrees of freedom and *P* value noted<br>*Give P values as exact values whenever suitable.*
☐	☒	For Bayesian analysis, information on the choice of priors and Markov chain Monte Carlo settings
☒	☐	For hierarchical and complex designs, identification of the appropriate level for tests and full reporting of outcomes
☐	☒	Estimates of effect sizes (e.g. Cohen's *d*, Pearson's *r*), indicating how they were calculated

Our web collection on statistics for biologists contains articles on many of the points above.

Software and code

Policy information about availability of computer code

Data collection	Data from the UK Biobank are publicly available after approval. Stefano Romeo has approval to access the data with Application Number 37142. All the phenotypic and genetic data used in this study were retrieved from official data-fields available on the UK Biobank and imported in MATLAB R2024b (Mathworks, https://www.mathworks.com/) or R v4.0.2 (https://www.r-project.org/) for further data preparation before the main analyses.<br>MRI image handling and segmentation used the UK Biobank abdominal MRI preprocessing pipeline (Software Heritage revision swh:1:rev:13dc77941cb2919417108637eade6c8448374229) and ukbb-mri-sseg (Software Heritage revision swh:1:rev:4acdad6bf5e6cd08436d91ac6d4a494cf1365d98).

Data analysis	All code and scripts used for the analyses are available at https://github.com/Ojami/PDFF-META-GWAS and archived on Zenodo (https://doi.org/10.5281/zenodo.22672918). Analyses were performed using the MAtlab GEnetics Toolbox (MAGE v1.0.0: https://github.com/Ojami/MAGE). MATLAB R2024b was used under an academic license. Publicly available tools used in this work are listed as below: METAL version "2020-05-05": https://genome.sph.umich.edu/wiki/METAL. REGENIE v4.1: https://github.com/rgcgithub/regenie. PLINK v1.90b6.26: https://www.cog-genomics.org/plink/. GCTA-COJO v1.94.0: https://yanglab.westlake.edu.cn/software/gcta/#COJO. LDSC v1.0.1: https://github.com/bulik/ldsc/. PolyFun v1.0.0: https://github.com/omerwe/polyfun. SuSiE v0.14.2: https://github.com/stephenslab/susieR. rtracklayer v1.54.0: https://bioconductor.org/packages/release/bioc/html/rtracklayer.html. coloc v5.2.3: https://github.com/chr1swallace/coloc. Ensembl Variant Effect Predictor (VEP) REST API: https://rest.ensembl.org/. FUMA v1.5.4 and MAGMA v1.08: https://fuma.ctglab.nl/. LDproxy: https://ldlink.nih.gov/?tab=ldproxy. R v4.0.2: https://www.r-project.org/. FLAMES v1.1.2: https://github.com/Marijn-Schipper/FLAMES. CALDERA: https://github.com/kheilbron/caldera. PoPS v0.2: https://github.com/FinucaneLab/pops. tensorQTL v1.0.9: https://github.com/broadinstitute/tensorqtl. FastQC v0.12.0; Trimmomatic v0.39; STAR v2.7.10a; RSEM v1.3.3; GCTB v2.5.2 (for SBayesRC); PLINK v2.0; GSEApy v1.0.5; and MAGMA v1.10.

For manuscripts utilizing custom algorithms or software that are central to the research but not yet described in published literature, software must be made available to editors and reviewers. We strongly encourage code deposition in a community repository (e.g. GitHub). See the Nature Portfolio guidelines for submitting code & software for further information.

Data

Policy information about availability of data

All manuscripts must include a data availability statement. This statement should provide the following information, where applicable:

- Accession codes, unique identifiers, or web links for publicly available datasets
- A description of any restrictions on data availability
- For clinical datasets or third party data, please ensure that the statement adheres to our policy

All data associated with this study are present in the paper or the Supplementary Information. The PDFF meta-analysis summary statistics have been deposited in the NHGRI-EBI GWAS Catalog under accession GCST90994130. For UK Biobank, all individual-level phenotype/genotype data are accessible via a formal application to the UK Biobank http://www.ukbiobank.ac.uk. The ethical approval of the MAFALDA study restricts the public sharing of individual data. However, researchers may submit a proposal to access liver biopsy data from the MAFALDA cohort, either in raw or analyzed form, between 9 and 36 months after publication. Proposals should be directed to Stefano Romeo at stefano.romeo@ki.se. Stefano Romeo will review each request to assess data availability. Responses will be provided within 8 weeks of receiving the request. It's important to note that patient-related data may be restricted due to confidentiality regulations. If approved for sharing, data will be transferred under a material transfer agreement. The following online databases have been used: GWAS catalog, https://www.ebi.ac.uk/gwas/; baseline LD model: https://data.broadinstitute.org/alkesgroup/LDSCORE/. Summary statistics for MVP are available under dbGaP study accession phs002453.v1.p1 https://ftp.ncbi.nlm.nih.gov/dbgap/studies/phs002453/analyses/HARE/. FinnGen GWAS summary statistics are publicly accessible following registration (https://www.finngen.fi/en/access_results). Summary statistics for deCODE and Intermountain are publicly accessible following registration (https://www.decode.com/summarydata/).

Research involving human participants, their data, or biological material

Policy information about studies with human participants or human data. See also policy information about sex, gender (identity/presentation), and sexual orientation and race, ethnicity and racism.

Reporting on sex and gender	In this study, sex was determined based on central registry at recruitment (from NHS) and self-reporting (https://biobank.ndph.ox.ac.uk/showcase/field.cgi?id=31). All our analyses were adjusted for sex. Thus, our findings are independent from sex and apply to both sexes. In phenome-wide association analyses using UKB Phecode v1.2b1 ICD mapping, for sex-specific phecodes, individuals of the opposite sex were also excluded
Reporting on race, ethnicity, or other socially relevant groupings	In this study, we used the European subset of UK Biobank individuals by including the individuals self-reported as "Irish" or "any other White background", and further removed outliers based on first 6 genetic principal components of ancestry. These individuals along with the subset of participants of White British ancestry were considered as Europeans. Participants from replication cohorts were self-reported as European ancestry (MAFALDA cohort). Confounding variables were accounted for by adjusting the regression models for age, sex, indices of adiposity, first 10 genomic principal components and array batch. The confounding bias in the GWA studies were further evaluated using LDSC software (https://github.com/bulik/ldsc/).
Population characteristics	The MRI-derived PDFF analyses included 71,488 participants of European ancestry. Association analyses were adjusted for age at MRI, sex, age², age×sex, age²×sex, BMI at MRI, genotyping array, and the first ten principal components of ancestry. Liver cis-eQTL mapping was performed in 217 MAFALDA participants with morbid obesity and available liver bulk RNA-seq and genotype data; the model included ten genetic principal components and 35 expression principal components.
Recruitment	The UK Biobank is a population-based study which has recruited over 500,000 participants aged between 40 and 69 years across the UK between 2006 and 2010, with extensive phenotypic and genetic data. In this study, only individuals of European ancestry were included. Additionally, subjects were excluded if they fall into any of these categories: 1) more than 10 putative 3rd degree relatives, 2) a mismatch between self-reported and genetically inferred sex, 3) putative sex chromosome aneuploidy, 4) heterozygosity and missingness outliers, and 5) withdrawn consent.<br>The MAFALDA comprises a total of 468 consecutive participants with morbid obesity (BMI ≥35 kg/m2) that underwent bariatric surgery at Campus Bio-Medico University of Rome, Italy in whom SLD diagnosis was assessed by liver histology or vibration-controlled transient elastography including CAP measurement with FibroScan® (Echosens, Paris, France). In the present study, only individuals with available bulk RNA-seq for liver and genotyping data were included.
Ethics oversight	The UK Biobank received ethical approval from the National Research Ethics Service Committee Northwest Multi-Centre

Ethics oversight	Haydock (reference 16/NW/0274). Data used in this study were obtained under application number 37142. The MAFALDA study has been approved by the Local Research Ethics Committee (no. 16/20).

Note that full information on the approval of the study protocol must also be provided in the manuscript.

Field-specific reporting

Please select the one below that is the best fit for your research. If you are not sure, read the appropriate sections before making your selection.

☒ Life sciences ☐ Behavioural & social sciences ☐ Ecological, evolutionary & environmental sciences

For a reference copy of the document with all sections, see nature.com/documents/nr-reporting-summary-flat.pdf

Life sciences study design

All studies must disclose on these points even when the disclosure is negative.

Sample size	No statistical method was used to predetermine sample size. We included the three non-overlapping MRI-derived PDFF datasets available in the UK Biobank after quality control and removal of overlapping participants: gradient-echo PDFF (n = 1,200), IDEAL-derived PDFF measured in this study (n = 35,179), and Perspectum-derived PDFF (n = 35,109). The resulting sample of 71,488 participants represents the largest genome-wide association study of MRI-derived liver fat content to date and was sufficient to identify 60 genome-wide significant loci and estimate SNP-based heritability.
Data exclusions	In the UK Biobank, subjects were excluded if they fall into any of these categories: 1) more than 10 putative 3rd degree relatives, 2) a mismatch between self-reported and genetically inferred sex, 3) putative sex chromosome aneuploidy, 4) heterozygosity and missingness outliers, and 5) withdrawn consent. For all the study cohorts, individuals of self-reported non-European ancestry or with genotype/phenotype unavailable data were excluded. The exclusion criteria were decided a priori.
Replication	The 31 variants not previously reported for liver triglyceride content were evaluated in a meta-analysis of clinically diagnosed MASLD comprising 42,163 cases and 1,646,254 controls across five European-ancestry studies (deCODE, Intermountain, FinnGen, MVP and UK Biobank). UK Biobank participants with available PDFF measurements (n = 71,488) were excluded before the meta-analysis to prevent sample overlap.
Randomization	This is a genetic association study. The study groups are defined based on the genotype and randomized by nature.
Blinding	This was no intervention study thus blinding was not relevant.

Reporting for specific materials, systems and methods

We require information from authors about some types of materials, experimental systems and methods used in many studies. Here, indicate whether each material, system or method listed is relevant to your study. If you are not sure if a list item applies to your research, read the appropriate section before selecting a response.

Materials & experimental systems

n/a	Involved in the study	
☒	☐	Antibodies
☒	☐	Eukaryotic cell lines
☒	☐	Palaeontology and archaeology
☒	☐	Animals and other organisms
☐	☒	Clinical data
☒	☐	Dual use research of concern
☒	☐	Plants

Methods

n/a	Involved in the study	
☒	☐	ChIP-seq
☒	☐	Flow cytometry
☒	☐	MRI-based neuroimaging

Clinical data

Policy information about clinical studies

All manuscripts should comply with the ICMJE guidelines for publication of clinical research and a completed CONSORT checklist must be included with all submissions.

Clinical trial registration	this was not a clinical trial
Study protocol	this was not a clinical trial
Data collection	this was not a clinical trial
Outcomes	this was not a clinical trial

Plants

Seed stocks	NA
Novel plant genotypes	NA
Authentication	NA
