# Supplementary material for: Genome-wide analyses identify 60 loci associated with liver triglyceride content
Source: Nat Genet. 2026 Oct 21;58(11):2772–7. doi: 10.1038/s41588-026-02794-6 (PMC13754591; doi:10.1038/s41588-026-02794-6)
Supplement: Supplementary file 3 [file 41588_2026_2794_MOESM3_ESM.pdf]

Peer Review File

Genome-wide analyses identify 60 loci associated with liver triglyceride content

Corresponding Author: Professor Stefano Romeo

Version 0:

Decision Letter:

23rd March 2026

Dear Stefano,

Thank you for inquiring about our interest in this proposed submission. It sounds interesting and we would like to see it, although I am sure you will appreciate that it can be very difficult to judge a paper without having seen it in its entirety. We cannot guarantee that we will send it out for review until we have had a chance to assess the full manuscript.

Please note that supplementary materials must not be included with the main text and figures of your manuscript. Supplementary items should be uploaded as separate files.

At this stage, you will also need to upload a completed Reporting Summary:

https://www.nature.com/documents/nr-reporting-summary.pdf

(Here you can read about the role of the Reporting Summary in reproducible science:
https://www.nature.com/news/announcement-towards-greater-reproducibility-for-life-sciences-research-in-nature-1.22062)

In order to submit your complete manuscript, please use the link below and follow the prompt to “Revise NG-PI72345”:

Link Redacted

We look forward to receiving it.

Sincerely,
Kyle

Kyle Vogan, PhD
Senior Editor
Nature Genetics
https://orcid.org/0000-0001-9565-9665

Version 1:

Decision Letter:

27th May 2026

Dear Stefano,

Your Letter "Genome-wide association study identifies 60 loci associated with liver triglyceride content" has been seen by two referees. You will see from their comments below that, while they find your work of potential interest, they have raised substantial concerns that must be addressed. In light of these comments, we cannot accept the manuscript for publication at

this time, but we would be interested in considering a suitably revised version that addresses the referees' concerns.

We hope you will find the referees' comments useful as you decide how to proceed. If you wish to submit a substantially revised manuscript, please bear in mind that we will be reluctant to approach the referees again in the absence of major revisions.

To guide the scope of the revisions, the editors discuss the referee reports in detail within the team with a view to identifying key priorities that should be addressed in revision, and sometimes overruling referee requests that are deemed beyond the scope of the current study. In this case, we ask that you address all technical concerns related to the design and interpretation of the genetic association analyses (including treatment of phenotyping heterogeneity and important variables such as alcohol consumption), revise the presentation to further place the results in context with previous findings, and perform additional validation studies where feasible. We hope you will find this prioritized set of referee points to be useful when revising your study. Please do not hesitate to get in touch if you would like to discuss these issues further.

If you choose to revise your manuscript taking into account all reviewer and editor comments, please highlight all changes in the manuscript text file. At this stage, we will need you to upload a copy of the manuscript in MS Word .docx or similar editable format.

We are committed to providing a fair and constructive peer-review process. Do not hesitate to contact us if there are specific requests from the reviewers that you believe are technically impossible or unlikely to yield a meaningful outcome.

If revising your manuscript:

*1) Include a “Response to referees” document detailing, point-by-point, how you addressed each referee comment. If no action was taken to address a point, you must provide a compelling argument. This response will be sent back to the referees along with the revised manuscript.

*2) If you have not done so already, please begin to revise your manuscript so that it conforms to our Letter format instructions, available here.
Refer also to any guidelines provided in this letter.

*3) Include a revised version of your Reporting Summary: https://www.nature.com/documents/nr-reporting-summary.pdf
It will be available to referees (and, potentially, statisticians) to aid in their evaluation if the manuscript goes back for peer review.
A revised Reporting Summary is essential for re-review of the paper.

Please be aware of our guidelines on digital image standards.

EXTENDED DATA FIGURES

When re-submitting your manuscript, please ensure that any supplementary figures and tables that are crucial to the manuscript's conclusions are converted into Extended Data figures and tables to increase visibility of these data. Extended Data figures and tables are online-only (present in the online PDF and full-text HTML versions of the paper), peer-reviewed display items that provide essential background to the article but are not included in the main article due to space constraints. A maximum of ten Extended Data display items (figures and tables) is permitted.

You may use the link below to submit your revised manuscript and related files:

Link Redacted

Note: This URL links to your confidential home page and associated information about manuscripts you may have submitted, or that you are reviewing for us. If you wish to forward this email to co-authors, please delete the link to your homepage.

If you wish to submit a suitably revised manuscript, we hope to receive it within 3-6 months. If you cannot send it within this time, please let us know. We will be happy to consider your revision so long as nothing similar has been accepted for publication at Nature Genetics or published elsewhere. Should your manuscript be substantially delayed without notifying us in advance and your article is eventually published, the received date would be that of the revised, not the original, version.

Please do not hesitate to contact me if you have any questions or would like to discuss the required revisions further.

Nature Genetics is committed to improving transparency in authorship. As part of our efforts in this direction, we are now requesting that all authors identified as ‘corresponding author’ on published papers create and link their Open Researcher and Contributor Identifier (ORCID) with their account on the Manuscript Tracking System (MTS), prior to acceptance. ORCID helps the scientific community achieve unambiguous attribution of all scholarly contributions. You can create and link your ORCID from the home page of the MTS by clicking on ‘Modify my Springer Nature account’. For more information, please visit www.springernature.com/orcid.

Thank you for the opportunity to review your work.

Sincerely,
Kyle

Kyle Vogan, PhD
Senior Editor
Nature Genetics
https://orcid.org/0000-0001-9565-9665

Referee expertise:

Referee #1: Genetics, hepatic diseases

Referee #2: Genetics, hepatic diseases

Reviewers' Comments:

Reviewer #1 (Remarks to the Author):

Summary of the key results: This study presents the largest GWAS to date of MRI-derived liver triglyceride content (PDFF) in around 71,000 individuals from UKBB. The authors identify 60 genome-wide significant loci, including 31 reported as novel, and integrate these findings with gene prioritization, PRS analyses, phenome-wide analyses, and plasma proteomics. They report that genetic susceptibility to liver fat is associated with downstream metabolic pathways and increased risk of cirrhosis and HCC.

Originality and significance: The primary strength and novelty lie in the scale of the GWAS and the identification of additional loci. However, the overall novelty is moderate. Many downstream findings, particularly associations between genetic predisposition to liver fat and cardiometabolic disease, cirrhosis, and HCC, are largely confirmatory and consistent with prior literature (e.g., Sveinbjornsson et al., Nature Genetics 2022; Parisinos et al., J Hepatol 2020; Ghouse et al., Nat Genet 2024). The manuscript would benefit from a clearer distinction on what is novel.

Data & methodology: The study leverages high-quality UKB data and state of the art GWAS methodology. The large sample size is a major strength. However, several methodological concerns remain:

a. All discovery and most downstream analyses are confined to UK Biobank, limiting external validity.

b. The PDFF phenotype is derived from three imaging sources with different acquisition and processing pipelines, introducing potential measurement inconsistency.

c. The in-house PDFF extraction, while well correlated with external pipelines, relies on somewhat ad hoc QC procedures that are insufficiently characterized.

d. The exclusive focus on EUR ancestry limits generalizability and misses an opportunity for cross-ancestry validation.

Appropriate use of statistics and treatment of uncertainties: Statistical methods are generally appropriate and standard for the field.

Conclusions: The identification of loci associated with liver fat is robust. However, the broader conclusions are somewhat overstated:

a. Claims regarding biological mechanisms (e.g., inflammatory pathways, carcinogenesis vs. fibrogenesis) are based on associations and should be interpreted cautiously.

b. Gene prioritization remains tentative due to limited eQTL sample size and heuristic scoring approaches.

Suggested improvements:

Major concerns

1. While the identification of novel loci is a clear strength, the overall novelty of the downstream analyses is somewhat limited. Associations between polygenic risk for liver fat and progression to cirrhosis or HCC, as well as broader cardiometabolic phenotypes, are broadly consistent with prior literature. As such, the manuscript would benefit from more clearly delineating what is genuinely new versus confirmatory, particularly in the PRS, PheWAS, and proteomic analyses.

2. The entire discovery analysis is restricted to EUR-ancestry participants from UKB, and the authors themselves

acknowledge this as a limitation. This is a major issue for a genetics paper proposing a broader map of liver triglyceride biology, especially given known ancestry differences in allele frequencies, LD structure, and PRS portability. Given that there appear to be around 1-2k AFR and SAS individuals available, it would be worthwhile to explore these data, at least descriptively, to make the study more broadly informative.

3. Although the three PDFF datasets are non-overlapping, all discovery data come from UKB. The PRS analyses, PheWAS, and proteomics are also performed in UKB after excluding PDFF participants. This reduces the extent of true external validation and raises concern that the downstream findings may largely reflect one cohort's structure, ascertainment, and coding practices rather than broadly transportable biology.

4. The paper combines three different PDFF sources with different imaging acquisitions and derivation methods, then meta-analyzes them. The authors perform a sensitivity harmonization analysis, which is reassuring, but the core phenotype remains heterogeneous. One source is very small (around 1.2k), one is extracted by the authors, and one is Perspectum-derived. This creates room for measurement non-equivalence and residual heterogeneity that a fixed-effect meta-analysis may not fully capture.

5. The in-house PDFF extraction appears technically strong, but the QC strategy still seems somewhat ad hoc. Outlier exclusion is based on a single feature, an Isolation Forest threshold, and "provisional outliers" defined against another UKB field. Only 188 scans were removed, but it remains difficult to judge whether residual mismeasurement persists, especially when this custom-derived phenotype contributes roughly half the sample. Additional visualization and error characterization would strengthen confidence.

6. The GWAS, PRS analyses, PheWAS, and proteomics all adjust for BMI. This is understandable if the aim is to isolate liver-specific fat biology, but BMI may also lie on the causal pathway or share substantial genetic architecture with PDFF. This could attenuate biologically relevant signals or complicate interpretation of loci that jointly influence adiposity and hepatic fat. The manuscript does not sufficiently discuss this estimand issue.

7. Of the 31 newly reported variants, only 14 pass Bonferroni correction in the MASLD meta-analysis, while 22 are nominally significant. This is respectable, but weaker than the framing may suggest. In addition, the validation phenotype is clinically diagnosed MASLD based on ICD coding across multiple studies, which is a noisier and biologically different phenotype from MRI-derived PDFF. It would be helpful to know how comparable these MASLD definitions are across contributing cohorts such as MVP vs. FinnGen. Genetic correlation analyses across studies or phenotypes would strengthen the interpretation.

8. Cirrhosis and HCC are identified from ICD codes, and viral hepatitis is excluded, but there is little discussion of competing etiologies such as alcohol-related liver disease, autoimmune liver disease, cholestatic liver disease, or general misclassification in registry-based endpoints. For a paper making claims about progression from liver triglyceride content to end-stage liver disease, endpoint specificity is very important.

9. Several interpretations go beyond what the data strictly support. For example, the proteomics section states that this is the "first genetic evidence implicating a specific inflammatory pathway in lipid-mediated hepatocyte damage," and the Discussion suggests that susceptibility to hepatic lipid accumulation is "more tightly linked to carcinogenesis than to fibrogenesis." These are interesting hypotheses, but the analyses are associative and largely PRS-based. The wording should therefore be toned down. In the same vein, PRS analyses contrast the highest decile with the lowest decile, which are two extremes. It would be more informative to report effect estimates relative to the median decile as well.

10. The proteomics are associated with a genome-wide PRS rather than with measured PDFF itself, and many loci contributing to a genome-wide score may act through pathways that are not specific to liver fat. The authors partly acknowledge this in the contrast between the genome-wide PRS and the 60-variant PRS in the PheWAS, but the same limitation applies to the proteomic pathway enrichment. Some of the signal may reflect general cardiometabolic liability rather than liver-triglyceride-specific biology.

11. The paper integrates FLAMES, CALDERA, and liver cis-eQTL colocalization into a summed "confidence score". This is pragmatic, but still quite arbitrary. The thresholds are chosen by the authors, the eQTL data come from only 217 liver samples, and the fallback rules allow a top-ranked gene to be retained even when none of the main confidence criteria are met. As a result, the 48 "supported candidate effector genes" should be presented more explicitly as tentative rather than near-resolved. Relatedly, it is not clear why GTEx liver eQTL data were not also used, given the larger sample size.

12. Given the clear phenotype differences between datasets, a fixed-effect inverse-variance model may understate uncertainty if there is genuine between-dataset heterogeneity. I would like to see heterogeneity statistics for the lead loci reported more clearly across datasets.
Minor or moderate concerns

13. The gradient-echo dataset includes only 1,200 participants, which is much smaller than the two main datasets. It likely contributes limited information while adding additional phenotypic heterogeneity.

14. The abstract states that 60 loci explain 25% of the heritability, whereas the main text reports SNP-based heritability of PDFF as 25%. This wording is potentially confusing unless the manuscript clearly distinguishes variance explained by the identified loci from total SNP heritability.

15. There are substantial industry relationships and patent/equity disclosures for senior authors. This does not invalidate the work, but for a translational genetics paper emphasizing PRS and biomarker implications, it reinforces the need to avoid overstatement of clinical utility.

References: Key prior studies are cited, but the manuscript tends to benchmark novelty primarily against the authors' previous work. Broader contextualization within the existing NAFLD/MASLD GWAS literature is needed. Some loci described as "novel" may have been implicated previously in related phenotypes (e.g., ERLIN1 in Sveinbjornsson et al., 2022), and this should be more transparently addressed.

Clarity and context: The manuscript is well written and structured.

Reviewer #2 (Remarks to the Author):

The article reports on a UK Biobank based GWAS on liver triglyceride content with associated validation versus independent clinically manifest MASLD GWAS datasets. The analytical flow appears standard, with potential criticism mainly pertaining to phenotype definitions, confounders and reporting. The article has several interesting features and adds to current literature on MASLD-related biology.

Some points warrant consideration:

1) There is no recognition of the spectrum of ALD-MetALD-MASLD in the approach taken, in particular relevant incorporation of alcohol consumption-related variables and several important cardiometabolic risk factors (CMRFs) in the analytical approach, beyond that pertaining to the PheWAS (see also comment 8 below) presented in Figure 3. See e.g. https://www.journal-of-hepatology.eu/article/S0168-8278(25)02326-8/fulltext and co-authors on the manuscript are aware of this. The complete omission of handling alcohol as a relevant co-variable is the most significant to this reviewer.

2) The lack of generalizability to other European is hampered by UK specifics of liver disease (see e.g. PMID 30060999), enhancing the importance of point 1 – and should be mentioned in the text.

3) Robustness of the 31 claimed novel associations appears slightly opaque; for some of these loci (not easy to read from the provided materials) there is independent support for a genuine association, e.g. the overlap for 14 of the loci with previous MASLD GWAS. Exactly for how many of the remaining 17 loci there is additional support (beyond the GWS p-values in the UK Biobank query) is not clear, and reporting of these loci must acknowledge the risk of type 1 errors.

4) I remain puzzled about the claim that data supports "a specific inflammatory pathways in lipid-mediated hepatocyte damage". What aspects of analyzed data pertains to "hepatocyte damage"? Were transaminases considered in the analytical pipeline? Fibrosis measures?

5) I would have expected to see a deeper cross-comparison of findings with past GWAS, beyond that pertaining to the PheWAS, similar to assessments made for MASLD, HCC and cirrhosis, i.e. including other CMRFs, and it could also have been interesting to see comparisons versus relevant blood test GWAS, including tryglycerides and transaminases.

6) I fully understand and agree to the exclusion of viral hepatitis (in both analyses? – page 13 – how many?) but would have found analyses on specific features to that end useful to expand on the topic into this subpopulation.

7) I am not sure Figure 2 contains sufficient information over and above that presented in the text to justify a separate display item; clarifications to point 3 above would be more useful (e.g. table).

8) In Figure 3, under "mental disorders", I find "Alcoholic liver damage". How come? To this reviewer, this is unacceptable (and the term "alcoholic" is no longer used in relation to liver disease, as co-authors of the manuscript would be aware).

Version 2:

Decision Letter:

31st July 2026

Dear Stefano,

Your revised Letter "Genome-wide association study identifies 60 loci associated with liver triglyceride content" has been seen by the original reviewers. You will see from their comments below that, while Reviewer #1 is satisfied and has no further requests, Reviewer #2 has raised a few ongoing concerns. We remain interested in the possibility of publishing your study in Nature Genetics, but we would like to see your response to these ongoing concerns in the form of a further revision before we make a final decision on publication.

In particular, we ask that you report alcohol-adjusted association results for each genome-wide significant locus and provide

a more extensive discussion of the limitations of the current study design as requested by Reviewer #2.

We therefore invite you to revise your manuscript again taking into account all reviewer and editor comments. Please highlight all changes in the manuscript text file. At this stage, we will need you to upload a copy of the manuscript in MS Word .docx or similar editable format.

We are committed to providing a fair and constructive peer-review process. Do not hesitate to contact us if there are specific requests from the reviewers that you believe are technically impossible or unlikely to yield a meaningful outcome.

When revising your manuscript:

*1) Include a “Response to referees” document detailing, point-by-point, how you addressed each referee comment. If no action was taken to address a point, you must provide a compelling argument. This response will be sent back to the referees along with the revised manuscript.

*2) If you have not done so already, please begin to revise your manuscript so that it conforms to our Letter format instructions, available
here.
Refer also to any guidelines provided in this letter.

*3) Include a revised version of your Reporting Summary: https://www.nature.com/documents/nr-reporting-summary.pdf
It will be available to referees (and, potentially, statisticians) to aid in their evaluation if the manuscript goes back for peer review.
A revised Reporting Summary is essential for re-review of the paper.

Please be aware of our guidelines on digital image standards.

EXTENDED DATA FIGURES

When re-submitting your manuscript, please ensure that any supplementary figures and tables that are crucial to the manuscript’s conclusions are converted into Extended Data figures and tables to increase visibility of these data. Extended Data figures and tables are online-only (present in the online PDF and full-text HTML versions of the paper), peer-reviewed display items that provide essential background to the article but are not included in the main article due to space constraints. A maximum of ten Extended Data display items (figures and tables) is permitted.

Please use the link below to submit your revised manuscript and related files:

Link Redacted

Note: This URL links to your confidential home page and associated information about manuscripts you may have submitted, or that you are reviewing for us. If you wish to forward this email to co-authors, please delete the link to your homepage.

We hope to receive your revised manuscript within 4-8 weeks. If you cannot send it within this time, please let us know.

Please do not hesitate to contact me if you have any questions or would like to discuss these revisions further.

Nature Genetics is committed to improving transparency in authorship. As part of our efforts in this direction, we are now requesting that all authors identified as ‘corresponding author’ on published papers create and link their Open Researcher and Contributor Identifier (ORCID) with their account on the Manuscript Tracking System (MTS), prior to acceptance. ORCID helps the scientific community achieve unambiguous attribution of all scholarly contributions. You can create and link your ORCID from the home page of the MTS by clicking on ‘Modify my Springer Nature account’. For more information, please visit www.springernature.com/orcid.

We look forward to seeing the revised manuscript and thank you for the opportunity to review your work.

Sincerely,
Kyle

Kyle Vogan, PhD
Senior Editor
Nature Genetics
https://orcid.org/0000-0001-9565-9665

Reviewers' Comments:

Reviewer #1 (Remarks to the Author):

Thank you for the thorough and constructive responses to my comments. The authors have addressed my concerns carefully and have made substantial revisions that have improved the overall presentation of the study. I am satisfied with the responses and revisions and have no further comments.

Reviewer #2 (Remarks to the Author):

The authors have made some effort in responding to reviewer requests. For alcohol-intake (my point #1), as a key co-variable, also for the individual associations, Supplementary Table 11 (btw: Supplementary Tables remain poorly formatted) does not respond to the request as pertaining to the individually claimed/reported associations, only to findings on a global level. Given the many marginal (individual variants) associations claimed (amongst the total claimed 31 newly identified variants), I would expect to be able to see how many of the individual associations are significantly driven by alcohol-intake (versus other metabolic pathways) towards facilitating liver triglyceride accumulation. Further, to respond to my criticism point #2, by including a mere/single sentence in the discussion, largely neglects the problem of multiple claimed novel loci (some marginal, some non-validated) that still appear to rely on analysis of the single UK-based cohort (as also pointed out by reviewer #1). These issues are not yet sufficiently clear to the reader.

Version 3:

Decision Letter:

Our ref: NG-LE72345R2

27th August 2026

Dear Stefano,

Your revised manuscript "Genome-wide association study identifies 60 loci associated with liver triglyceride content" (NG-LE72345R2) has been seen by Reviewer #2, who has no further requests, and therefore we will be happy in principle to publish your study in Nature Genetics as a Letter pending final revisions to comply with our editorial and formatting guidelines.

We are now performing detailed checks on your paper, and we will send you a checklist detailing our editorial and formatting requirements soon. Please do not upload the final materials or make any revisions until you receive this additional information from us.

Thank you again for your interest in Nature Genetics. Please do not hesitate to contact me if you have any questions.

Sincerely,
Kyle

Kyle Vogan, PhD
Senior Editor
Nature Genetics
https://orcid.org/0000-0001-9565-9665

INTERNAL USE ONLY AIP modules required: ["Clinical","Life Science"]

Open Access This Peer Review File is licensed under a Creative Commons Attribution 4.0 International License, which permits use, sharing, adaptation, distribution and reproduction in any medium or format, as long as you give appropriate credit to the original author(s) and the source, provide a link to the Creative Commons license, and indicate if changes were made.
In cases where reviewers are anonymous, credit should be given to 'Anonymous Referee' and the source.
The images or other third party material in this Peer Review File are included in the article's Creative Commons license, unless indicated otherwise in a credit line to the material. If material is not included in the article's Creative Commons license and your intended use is not permitted by statutory regulation or exceeds the permitted use, you will need to obtain permission directly from the copyright holder.
To view a copy of this license, visit https://creativecommons.org/licenses/by/4.0/

Reviewer #1 (Remarks to the Author):

Summary of the key results: This study presents the largest GWAS to date of MRI-derived liver triglyceride content (PDFF) in around 71,000 individuals from UKBB. The authors identify 60 genome-wide significant loci, including 31 reported as novel, and integrate these findings with gene prioritization, PRS analyses, phenome-wide analyses, and plasma proteomics. They report that genetic susceptibility to liver fat is associated with downstream metabolic pathways and increased risk of cirrhosis and HCC.

Originality and significance: The primary strength and novelty lie in the scale of the GWAS and the identification of additional loci. However, the overall novelty is moderate. Many downstream findings, particularly associations between genetic predisposition to liver fat and cardiometabolic disease, cirrhosis, and HCC, are largely confirmatory and consistent with prior literature (e.g., Sveinbjornsson et al., Nature Genetics 2022; Parisinos et al., J Hepatol 2020; Ghouse et al., Nat Genet 2024). The manuscript would benefit from a clearer distinction on what is novel.

Data & methodology: The study leverages high-quality UKB data and state of the art GWAS methodology. The large sample size is a major strength. However, several methodological concerns remain:

a. All discovery and most downstream analyses are confined to UK Biobank, limiting external validity.

b. The PDFF phenotype is derived from three imaging sources with different acquisition and processing pipelines, introducing potential measurement inconsistency.

c. The in-house PDFF extraction, while well correlated with external pipelines, relies on somewhat ad hoc QC procedures that are insufficiently characterized.

d. The exclusive focus on EUR ancestry limits generalizability and misses an opportunity for cross-ancestry validation.

Appropriate use of statistics and treatment of uncertainties: Statistical methods are generally appropriate and standard for the field.

Conclusions: The identification of loci associated with liver fat is robust. However, the broader conclusions are somewhat overstated:

a. Claims regarding biological mechanisms (e.g., inflammatory pathways, carcinogenesis vs. fibrogenesis) are based on associations and should be interpreted cautiously.

b. Gene prioritization remains tentative due to limited eQTL sample size and heuristic scoring approaches.

Suggested improvements:

Major concerns

1. While the identification of novel loci is a clear strength, the overall novelty of the downstream analyses is somewhat limited. Associations between polygenic risk for liver fat and progression to cirrhosis or HCC, as well as broader cardiometabolic phenotypes, are broadly consistent with prior literature. As such, the manuscript would benefit from more clearly delineating what is genuinely new versus confirmatory, particularly in the PRS, PheWAS, and proteomic analyses.

We agree that the manuscript should more clearly separate the novelty of the genetic discovery from the downstream analyses used to interpret those findings. We have revised the Discussion to make this distinction explicit. The locus discovery, heritability estimate, gene prioritization analyses and MASLD validation are now presented as the main findings of the study, whereas the PRS, phenome-wide association and plasma protein analyses are framed as characterization of genetic susceptibility to liver triglyceride content in relation to clinical and molecular phenotypes. The revised Discussion now reads:

"Validation of 14 of the 31 previously unreported variants in clinically diagnosed MASLD indicates that MRI-derived liver triglyceride content captures inherited susceptibility relevant to clinical liver disease. The PRS, phenome-wide association and plasma protein analyses further characterized this genetic susceptibility through associations with advanced liver disease, cardiometabolic phenotypes and liver-enriched circulating proteins."

We also revised the concluding sentence to avoid overstating the novelty of the downstream analyses:

"In conclusion, this study identifies additional loci and candidate effector genes for liver triglyceride content and characterizes their associations with MASLD, cardiometabolic phenotypes, cirrhosis and HCC."

2. The entire discovery analysis is restricted to EUR-ancestry participants from UKB, and the authors themselves acknowledge this as a limitation. This is a major issue for a genetics paper proposing a broader map of liver triglyceride biology, especially given known ancestry differences in allele frequencies, LD structure, and PRS portability. Given that there appear to be around 1-2k AFR and SAS individuals available, it would be worthwhile to explore these data, at least descriptively, to make the study more broadly informative.

We agree that ancestry-specific analyses would make the study more informative. We had planned to perform PDFF association analyses in other ancestries in the UK Biobank, including African and South Asian participants. However, this analysis is not currently feasible because access to the UK Biobank Research Analysis Platform was suspended in April 2026 (https://community.ukbiobank.ac.uk/hc/en-gb/community/posts/36090225990173-Ongoing-closure-of-the-UKB-RAP). UK Biobank has announced phased reopening from September 2026, but result downloads will not be available initially. Limited manual output checking is planned later in 2026, and automated output checking is expected in early 2027 (https://community.ukbiobank.ac.uk/hc/en-gb/articles/37284566277789-Will-the-UKB-RAP-be-different).

However, we examined the 60 PDFF lead variants in the Dallas Heart Study (DHS), including PDFF associations in African, European and Hispanic participants, as well as a cross-ancestry meta-analysis. Effects in the figure below are aligned to the PDFF-increasing allele from our discovery GWAS. Not all variants were available after exact or proxy lookup. The strongest signals in DHS were at established PDFF *loci*: only *PNPLA3* and *TM6SF2* reached Bonferroni significance at $P < 0.05/60$. Nominally significant concordant associations were observed for 11 of 57 variants in the cross-

ancestry DHS analysis, 7 of 56 in African participants, 6 of 57 in European participants, and 2 of 55 in Hispanic participants.

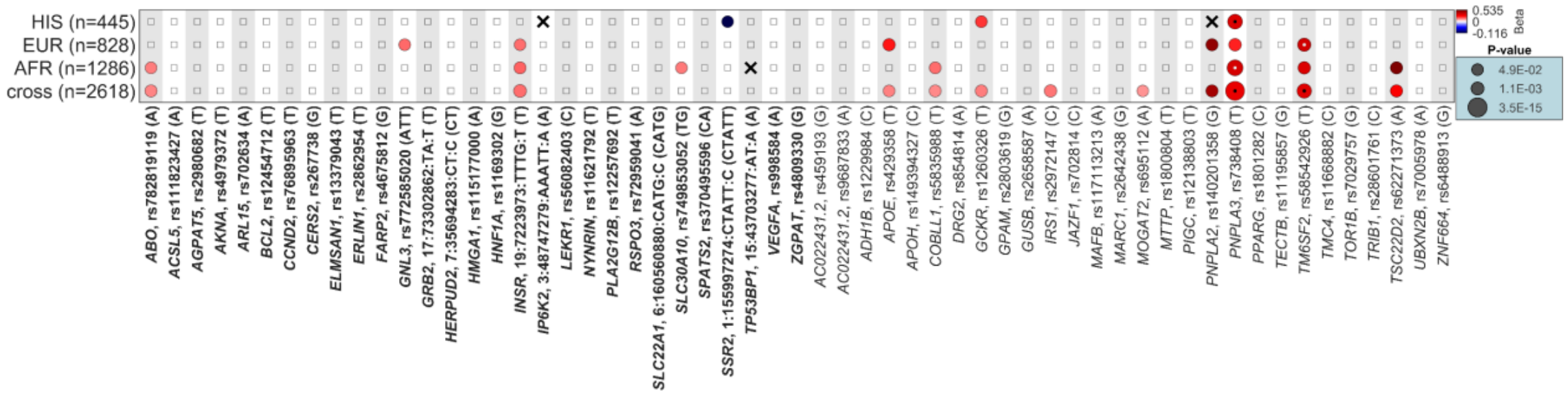

Dot plot showing DHS associations of the 60 PDFF lead variants in African, European and Hispanic participants, and in the cross-ancestry DHS meta-analysis. Effects are aligned to the PDFF-increasing allele from the discovery GWAS. Central marks indicate associations at P < 0.05/60. Crosses denote variants unavailable in the corresponding ancestry after exact or proxy lookup. Variant labels show the nearest gene, lead variant and PDFF-increasing allele, with newly identified loci shown in bold.

These results are consistent with low statistical power due to the smaller sample sizes available for PDFF in DHS. Even several established PDFF *loci* did not reach nominal significance in DHS ancestry-specific analyses, whereas the strongest known *loci* showed the clearest evidence. We therefore present the DHS results descriptively in this response, but have not added them as a main manuscript result. We have further clarified the limitation by noting that the discovery GWAS was restricted to participants of European ancestry and that larger MRI-derived PDFF studies in non-European ancestries are needed.

3. Although the three PDFF datasets are non-overlapping, all discovery data come from UKB. The PRS analyses, PheWAS, and proteomics are also performed in UKB after excluding PDFF participants. This reduces the extent of true external validation and raises concern that the downstream findings may largely reflect one cohort's structure, ascertainment, and coding practices rather than broadly transportable biology.

We agree that analyses performed in UK Biobank after excluding PDFF participants are not equivalent to an external validation. However, the PRS, PheWAS and plasma proteomic analyses were not intended as external validation of the discovery *loci*; they were used to characterize clinical and molecular associations of genetic susceptibility to liver triglyceride content. For external support of the newly identified *loci*, we used clinically diagnosed MASLD meta-analysis across multiple cohorts. To test whether the MASLD validation was driven by the UK Biobank study, we repeated the validation after excluding UK Biobank from the MASLD meta-analysis. The overall pattern was largely unchanged. In the full MASLD meta-analysis, 14 of 31 variants were Bonferroni-significant and concordant, and 22 were nominally significant and concordant. After excluding UK Biobank, 11 variants remained Bonferroni-significant and concordant, and 21 remained nominally significant and concordant. The three *loci* that no longer met Bonferroni significance were *CERS2* ($P = 9.97 \times 10^{-5}$ to $P = 0.0022$), *FARP2* ($P = 6.25 \times 10^{-4}$ to $P = 0.0062$), and *ARL15* ($P = 0.0012$ to $P = 0.0062$); all three retained concordant nominal associations. *VEGFA* no longer reached nominal significance ($P = 0.024$ to $P = 0.071$) but remained directionally concordant. Across the 31 variants, effect estimates were highly similar between the full and no-UK Biobank MASLD meta-analyses (Pearson R for beta = 0.97, and for Z score of association = 0.99). Taken all this together, data show that the MASLD validation was not driven by UK Biobank-specific coding or ascertainment.

4. The paper combines three different PDFF sources with different imaging acquisitions and

derivation methods, then meta-analyzes them. The authors perform a sensitivity harmonization analysis, which is reassuring, but the core phenotype remains heterogeneous. One source is very small (around 1.2k), one is extracted by the authors, and one is Perspectum-derived. This creates room for measurement non-equivalence and residual heterogeneity that a fixed-effect meta-analysis may not fully capture.

We thank the reviewer for raising this very important point. We agree that the three PDFF sources differ in acquisition and derivation, and we therefore analysed them as separate GWAS datasets before meta-analysis. The discovery GWAS used three non-overlapping sources: UK Biobank data-field 21092 from the single-slice gradient-echo acquisition, IDEAL-derived PDFF extracted in this work, and the Perspectum-derived PDFF (data-field 40061). To assess whether these sources capture the same genetic phenotype, we performed pairwise bivariate LDSC genetic correlation analyses across the PDFF GWAS, as shown in the table below. Because the non-overlapping data-field 21092 GWAS included only 1,200 participants and was imprecise for rg estimation, we also ran a full-sample data-field 21092 GWAS as a sensitivity analysis only for this validation step; this GWAS was not used in the discovery meta-analysis. Pairwise rg estimates were close to one, supporting high genetic concordance between the PDFF sources.

PDFF source 1	PDFF source 2	N1	N2	Sample overlap, N	rg	SE	P	gcov_int	gcov_int_SE
data-field 21092	IDEAL-derived	1200	35179	0	0.82	0.55	0.14	0.009	0.006
data-field 21092	Perspectum-derived	1200	35109	0	1.03	0.65	0.11	0.011	0.005
IDEAL-derived	Perspectum-derived	35179	35109	0	1.18	0.09	8.14E-37	0.033	0.015
IDEAL-derived	data-field 21092 (full)	35179	9056	74	1.11	0.19	2.53E-09	0.018	0.009
Perspectum-derived	data-field 21092 (full)	35109	9056	7782	1.00	0.10	1.15E-22	0.524	0.009

We report gcov_int and sample overlap for transparency. The elevated gcov_int in the full-sample data-field 21092 versus Perspectum-derived PDFF comparison is consistent with the substantial participant overlap in that sensitivity comparison. Values of rg slightly above one were interpreted with their standard errors as consistent with complete genetic correlation.

In addition, the meta-analysis did not pool raw PDFF values across sources. Each PDFF source was inverse-normal transformed and analysed separately, and the resulting association estimates were then combined by inverse-variance meta-analysis. This reduces the impact of differences in PDFF scale or distribution between acquisition and processing pipelines. As further sensitivity analyses, we quantified heterogeneity for the 60 identified lead variants across the three GWAS datasets and repeated the meta-analysis after excluding the small data-field 21092 GWAS. These analyses are described in response to comments 12 and 13. Briefly, the effects were highly concordant between the two larger PDFF sources, and exclusion of data-field 21092 did not substantially change the association estimates.

5. The in-house PDFF extraction appears technically strong, but the QC strategy still seems somewhat ad hoc. Outlier exclusion is based on a single feature, an Isolation Forest threshold, and “provisional outliers” defined against another UKB field. Only 188 scans were removed, but it remains difficult to judge whether residual mismeasurement persists, especially when this custom-derived phenotype contributes roughly half the sample. Additional visualization and error characterization would strengthen confidence.
We completely agree that the original description did not provide enough detail to judge the quality-control procedure or the extent of residual measurement error. We have expanded the Methods to describe the image-level QC measures that were evaluated, including summaries of the PDFF voxel

distribution and liver-mask boundary. We also clarify that the final filter was not chosen from a single pre-specified feature alone: we tested several QC summaries and five anomaly-detection approaches, and selected the Isolation Forest model using the 99th percentile of PDFF voxel values because it resulted in the highest cross-validated precision while flagging only a small proportion of scans. We added a new supplementary figure showing IDEAL-derived PDFF against data-field 24352 with excluded scans highlighted, and new supplementary tables quantifying agreement and absolute error before and after QC. These analyses show that scans removed by the QC filter had much larger differences from data-field 24352 than retained scans, whereas retained scans showed high agreement and modest residual absolute error. We also report agreement of the retained IDEAL-derived PDFF with the other PDFF sources used in the GWAS/meta-analysis (Supplementary Figure 1 and Supplementary Table 1).

6. The GWAS, PRS analyses, PheWAS, and proteomics all adjust for BMI. This is understandable if the aim is to isolate liver-specific fat biology, but BMI may also lie on the causal pathway or share substantial genetic architecture with PDFF. This could attenuate biologically relevant signals or complicate interpretation of loci that jointly influence adiposity and hepatic fat. The manuscript does not sufficiently discuss this estimand issue.

We agree that adjustment for BMI affects the interpretation of the GWAS and downstream analyses. Our primary aim in GWAS was to identify genetic associations with liver triglyceride content beyond generalized adiposity, rather than to estimate the total genetic effect on liver fat via BMI. We have now clarified this point in the Discussion. We also agree that variants acting mainly through BMI could be attenuated in a BMI-adjusted analysis. However, we believe BMI adjustment is justified for the primary analysis because genetically driven BMI increases MASLD risk, whereas genetically driven MASLD was not associated with generalized obesity in the reverse direction (PMID:32165250). In addition, genetic studies show that adiposity and liver fat can have discordant genetic effects: favourable adiposity alleles are associated with higher BMI and body fat but lower liver fat and lower cardiometabolic risk (PMID:30352878). As we showed previously, adiposity adjustment can also reveal liver fat *loci* where adiposity has inconsistent mediation or negative confounding effects (PMID:39653778). We therefore interpret the BMI-adjusted analyses as genetic associations with liver triglyceride content after accounting for generalized adiposity, and note that variants whose effects are mainly mediated through BMI may have attenuated associations. The revised Discussion now reads:

"Because BMI was included as a covariate, the GWAS and downstream analyses should be interpreted as genetic associations with liver triglyceride content that are not explained by generalized adiposity, although this may attenuate variants acting mainly through BMI."

7. Of the 31 newly reported variants, only 14 pass Bonferroni correction in the MASLD meta-analysis, while 22 are nominally significant. This is respectable, but weaker than the framing may suggest. In addition, the validation phenotype is clinically diagnosed MASLD based on ICD coding across multiple studies, which is a noisier and biologically different phenotype from MRI-derived PDFF. It would be helpful to know how comparable these MASLD definitions are across contributing cohorts such as MVP vs. FinnGen. Genetic correlation analyses across studies or phenotypes would strengthen the interpretation.

We agree that the MASLD validation phenotype required additional characterization because the contributing cohorts used related but not identical clinical definitions. We have now added a genome-wide genetic-correlation analysis between the PDFF GWAS meta-analysis and the clinically

diagnosed MASLD GWAS meta-analysis. PDFF and MASLD showed strong genetic correlation by bivariate LDSC (rg = 0.74, SE = 0.08, $P = 2.5 \times 10^{-21}$), supporting the use of MASLD as a validation phenotype for PDFF loci. This result has been added to the Results:

"To assess the genome-wide genetic overlap between MRI-derived liver triglyceride content and clinically diagnosed MASLD, we estimated their genetic correlation using bivariate LDSC. PDFF and MASLD showed a strong genetic correlation ($r_g = 0.74$, SE = 0.08, $P = 2.5 \times 10^{-21}$), supporting the use of MASLD as a validation phenotype for PDFF loci."

Additionally, because the MVP definition was broader than the other MASLD sources, we also assessed its genetic correlation with the two other sufficiently powered studies (i.e. UKBB and FinnGen). MVP showed high genetic correlation with FinnGen (rg = 0.85, SE = 0.13, $P = 2.6 \times 10^{-10}$) and UK Biobank (rg = 1.04, SE = 0.13, $P = 1.0 \times 10^{-15}$), and FinnGen and UK Biobank were also highly genetically correlated (rg = 1.07, SE = 0.19, $P = 2.7 \times 10^{-8}$). These analyses support broad genetic concordance across the main MASLD sources used for validation in our MASLD meta-analysis.

8. Cirrhosis and HCC are identified from ICD codes, and viral hepatitis is excluded, but there is little discussion of competing etiologies such as alcohol-related liver disease, autoimmune liver disease, cholestatic liver disease, or general misclassification in registry-based endpoints. For a paper making claims about progression from liver triglyceride content to end-stage liver disease, endpoint specificity is very important.

We agree that endpoint specificity is important for interpreting the association between genetic susceptibility to liver triglyceride content and end-stage liver disease. In the original analyses, chronic viral hepatitis was excluded, but other competing liver disease etiologies were not handled explicitly. We have now added sensitivity analyses for PRS-PDFF-GWAS in which cholestatic liver disease (ICD-10 K74.3, K74.4 and K74.5), autoimmune hepatitis (K75.4), alcohol-related liver disease (K70.0, K70.1, K70.2, K70.3, K70.4 and K70.9), and disorders of copper or iron metabolism (E83.0 and E83.1) were additionally excluded in case-control analyses and censored at diagnosis in prospective analyses.

The associations remained similar after applying these additional exclusion criteria. For example, per 1-s.d. increase in PRS-PDFF-GWAS, the association with cirrhosis was OR = 1.39/HR = 1.39 in the primary analysis and OR = 1.32/HR = 1.39 after applying the additional etiology exclusions. For HCC, the corresponding estimates were OR = 2.07/HR = 2.03 in the primary analysis and OR = 1.79/HR = 1.92 after applying the additional etiology exclusions. We also added sensitivity analyses adjusting for alcohol intake and excluding individuals above alcohol intake thresholds for metabolic dysfunction and alcohol-associated liver disease or alcohol-related liver disease. These results are now reported in Supplementary Table 11, and the Results have been revised to:

"Similar associations were observed in sensitivity analyses with additional adjustment for alcohol intake, exclusion of individuals above the alcohol intake thresholds for metabolic dysfunction and alcohol-associated liver disease or alcohol-related liver disease, or additional endpoint exclusions for competing liver disease etiologies (Supplementary Table 11)."

9. Several interpretations go beyond what the data strictly support. For example, the proteomics section states that this is the "first genetic evidence implicating a specific inflammatory pathway in lipid-mediated hepatocyte damage," and the Discussion suggests that susceptibility to hepatic lipid accumulation is "more tightly linked to carcinogenesis than to fibrogenesis." These are interesting hypotheses, but the analyses are associative and largely PRS-based. The wording should therefore be

toned down. In the same vein, PRS analyses contrast the highest decile with the lowest decile, which are two extremes. It would be more informative to report effect estimates relative to the median decile as well.

We thank the reviewer for this suggestion. We have revised the manuscript to avoid overinterpreting the PRS and proteomic analyses. Specifically, we removed the previous statement that the proteomic results provide "first genetic evidence" for a specific inflammatory pathway in lipid-mediated hepatocyte damage. We also revised the Discussion so that the comparison between cirrhosis and HCC is presented more carefully. We also revised the PRS decile analyses. In the original manuscript, the highest and lowest PRS deciles were compared only among the individuals in those two deciles. We now modelled all PRS deciles together as categorical predictors. Therefore, participants in the highest PRS decile had higher odds of cirrhosis (OR = 2.71, P = 1.61E-29) and HCC (OR = 7.47, P = 1.17E-14) compared with those in the lowest decile. To address the reviewer's request, we also repeated the decile analysis using the middle deciles of the PRS as the reference group. Compared with the middle deciles, the highest decile remained associated with higher odds of cirrhosis (OR = 2.01, P = 1.22E-27) and HCC (OR = 3.80, P = 9.52E-19). The full summary statistics are now shown in Supplementary Table 9, with corresponding effect plots in Supplementary Fig. 4, and the Results and Methods have been revised accordingly:

"In addition, participants in the highest PRS decile had markedly higher odds of cirrhosis (OR = 2.71, P= 1.61E-29) and hepatocellular carcinoma (OR = 7.47, P= 1.17E-14) compared to those in the lowest decile. Additionally, when compared with the middle deciles, the highest decile remained associated with higher odds of cirrhosis (OR = 2.01, P=1.22E-27) and hepatocellular carcinoma risk (OR = 3.80, P=9.52E-19). In prospective analyses, the PRS showed a similar risk of developing cirrhosis and hepatocellular carcinoma as observed in the case-control analyses (Supplementary Fig. 4; Supplementary Tables 9 and 10)."

10. The proteomics are associated with a genome-wide PRS rather than with measured PDFF itself, and many loci contributing to a genome-wide score may act through pathways that are not specific to liver fat. The authors partly acknowledge this in the contrast between the genome-wide PRS and the 60-variant PRS in the PheWAS, but the same limitation applies to the proteomic pathway enrichment. Some of the signal may reflect general cardiometabolic liability rather than liver-triglyceride-specific biology.

We thank the reviewer for raising this important comment, and we agree that the proteomic analyses based on the genome-wide PRS should not be interpreted as specific to liver triglyceride content, because a genome-wide score may capture broader cardiometabolic genetic liability. We have therefore revised the text and toned down mechanistic language. In particular, we compared PRS-PDFF-GWAS proteomic associations with those from the 60-variant PRS-PDFF. As shown below, across 2,923 proteins, the association Z scores were highly concordant between the two scores (Pearson r = 0.93). Of 750 proteins associated with PRS-PDFF at FDR < 0.05, 699 were also associated with PRS-PDFF-GWAS, and 99.7% of these shared proteins had concordant directions of effect. Thus, PRS-PDFF-GWAS retained the core proteomic signal observed with the 60-variant score.

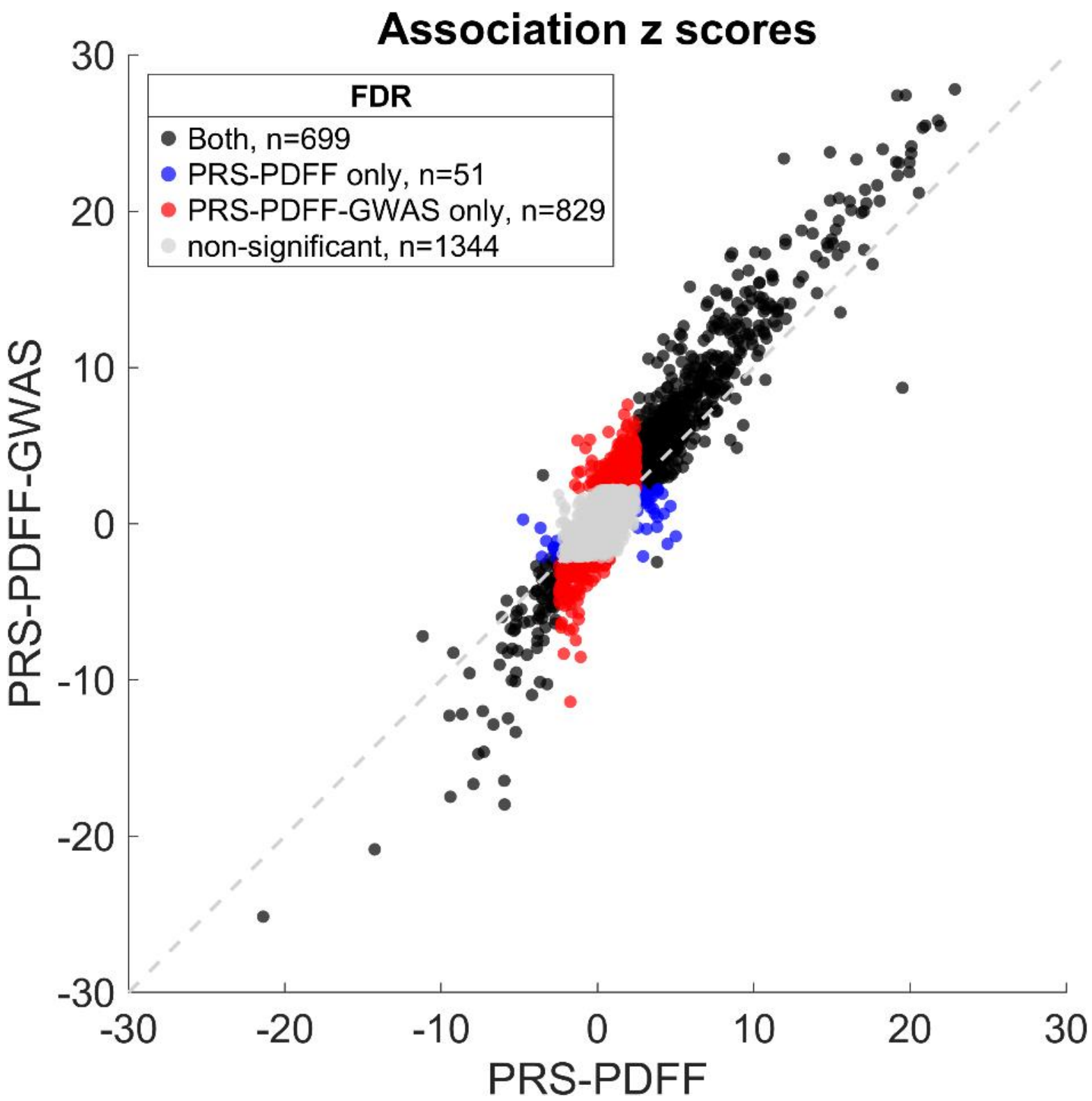

At the same time, PRS-PDFF-GWAS identified 829 additional FDR-significant proteins not detected with PRS-PDFF, suggesting that the genome-wide score may capture broader biology. We therefore added tissue-origin enrichment analyses comparing PRS-PDFF and PRS-PDFF-GWAS with relevant cardiometabolic PRSs. Liver-origin proteins were the top enriched tissue category for both PRS-PDFF and PRS-PDFF-GWAS, but PRS-PDFF showed the strongest liver-origin enrichment among the tested PRSs, whereas liver enrichment for PRS-PDFF-GWAS was similar to that observed for CAD PRS. We further performed MAGMA gene-property analysis using HPA cell type group annotations to examine cell-type enrichment in the PDFF GWAS signal. PDFF GWAS associations were enriched in both adipocyte- and hepatocyte-expressed genes. After excluding the fine-mapped intervals for the 60 *loci*, adipocyte enrichment remained significant, whereas hepatocyte enrichment was attenuated. These findings suggest that enrichment in adipocyte-expressed genes is broader in the genome-wide PDFF signal than in the 60-locus signal. We now report these analyses in the Results:

"To gain biological insight into the pathways associated with genetically predicted liver triglyceride content, we performed pre-ranked gene set enrichment analysis of the plasma proteins associated with PRS-PDFF-GWAS at false discovery rate below 0.05 in the UK Biobank. Reactome analysis showed enrichment for amino acid and carbohydrate metabolism, biological oxidations, sphingolipid metabolism and interleukin-10 signaling among proteins positively associated with higher genetic predisposition to liver triglyceride content. GTEx tissue enrichment showed a similar pattern, with proteins increasing with higher PRS enriched for liver expression, whereas proteins negatively associated with higher PRS were enriched for visceral adipose tissue expression (Supplementary Tables 13 and 14). We additionally performed a tissue-origin enrichment analysis using a plasma proteome distribution atlas and observed that liver-origin proteins were the top enriched tissue for both PRS-PDFF (OR = 3.03, FDR-adjusted P = 5.95×10^{-13}) and PRS-PDFF-GWAS (OR = 2.25, FDR-adjusted P = 4.04×10^{-7}). To assess whether this liver signal was specific to PDFF genetic susceptibility, we performed the same enrichment analysis for relevant cardiometabolic PRSs (see Methods). PRS-PDFF showed the strongest liver-origin enrichment among the PRSs tested, whereas liver enrichment for PRS-PDFF-GWAS was similar to that observed for CAD PRS (Supplementary Table 15). The inverse GTEx

enrichment for visceral adipose tissue expression is in line with recent evidence implicating reduced visceral adipocyte differentiation in MASLD. The enrichment of amino acid metabolism is notable given recent evidence that amino acids can serve as a major carbon source for hepatic lipogenesis. Interleukin-10 signaling was largely driven by pro-inflammatory cytokines and chemokines, including IL6, TNF and CXCL10. Together, these results highlight cytokine and chemokine signaling among the plasma protein changes associated with higher genetically predicted liver triglyceride content, consistent with prior work showing that these pathways are central to hepatic inflammation and MASLD progression.

To further examine cell-type enrichment in the PDFF GWAS signal, we performed MAGMA gene-property analysis using cell type group annotations from the Human Protein Atlas (HPA). PDFF GWAS associations were enriched in both adipocyte- and hepatocyte-expressed genes, with stronger enrichment in adipocytes. After excluding the fine-mapped intervals for the 60 loci, adipocyte enrichment remained significant, while hepatocyte enrichment was attenuated (Supplementary Table 16). These findings suggest that enrichment in adipocyte-expressed genes is broader in the genome-wide PDFF signal than in the 60-locus signal."

11. The paper integrates FLAMES, CALDERA, and liver cis-eQTL colocalization into a summed "confidence score". This is pragmatic, but still quite arbitrary. The thresholds are chosen by the authors, the eQTL data come from only 217 liver samples, and the fallback rules allow a top-ranked gene to be retained even when none of the main confidence criteria are met. As a result, the 48 "supported candidate effector genes" should be presented more explicitly as tentative rather than near-resolved. Relatedly, it is not clear why GTEx liver eQTL data were not also used, given the larger sample size.

We agree that the confidence score should be interpreted as a prioritization score rather than definitive resolution of causal genes. We have revised the manuscript accordingly and now refer to these genes as candidate effector genes. The score was used to combine complementary evidence from FLAMES, CALDERA and liver eQTL colocalization, with FLAMES providing a calibrated 75% precision threshold, CALDERA providing calibrated causal gene probabilities, and colocalization testing whether the GWAS and liver eQTL signals are compatible with a shared variant. We agree that the summed score and fallback rule are heuristic, and this is now stated more cautiously. We additionally repeated liver eQTL colocalization using GTEx v10. Although GTEx liver had a larger sample size, MAFALDA provided more colocalization signals at PP.H4 ≥ 0.8 (27 gene locus pairs across 20 *loci*, versus 15 gene locus pairs across 14 *loci* in GTEx). Ten GTEx signals overlapped MAFALDA results, including *MBOAT7*, *SLC25A19*, *GSTM5*, *ANXA9*, *AKNA* and *NYNRIN*, and the five GTEx only signals did not change the retained candidate genes. We therefore kept the original prioritization, while noting that MAFALDA provides liver eQTL data from individuals with obesity, a context relevant to SLD.

12. Given the clear phenotype differences between datasets, a fixed-effect inverse-variance model may understate uncertainty if there is genuine between-dataset heterogeneity. I would like to see heterogeneity statistics for the lead loci reported more clearly across datasets.

We agree that heterogeneity across the three PDFF sources should be reported more clearly. Supplementary Table 1 already included Cochran's Q heterogeneity P value and the direction of effect across the three GWAS datasets. We have now added the I^2 (%) statistic to the same table, so that both the statistical evidence for heterogeneity and the estimated proportion of between-source variability are shown for each lead variant. Overall, heterogeneity was not widespread among the 60 lead variants. Seven of 60 lead variants had $I^2 > 50\%$, and four had nominal heterogeneity $P < 0.05$.

However, no lead variant met a Bonferroni-corrected heterogeneity threshold of $P^{Het} < 8.3 \times 10^{-4}$ (0.05/60). We therefore retained the fixed-effect inverse-variance meta-analysis as the primary discovery analysis, while reporting I^2 and P^{Het} transparently for all lead variants.

Minor or moderate concerns

13. The gradient-echo dataset includes only 1,200 participants, which is much smaller than the two main datasets. It likely contributes limited information while adding additional phenotypic heterogeneity.

We agree that data-field 21092 is much smaller than the two main PDFF datasets and therefore contributes limited weight to the meta-analysis. To assess whether this dataset affected the discovery findings, we repeated the meta-analysis after excluding data-field 21092 and retaining only the IDEAL-derived and Perspectum-derived PDFF GWAS datasets. The sensitivity meta-analysis included 70,288 participants, compared with 71,488 in the primary analysis. As shown below, the lead-variant results were essentially unchanged. Across the 60 primary lead variants, effect estimates from the primary and sensitivity meta-analyses were almost identical (Pearson R = 0.9999), and the association Z scores were also highly concordant (Pearson R = 0.9999). All 60 variants had the same direction of effect. Fifty eight of 60 variants remained genome-wide significant after excluding data-field 21092; the two variants that no longer met $P < 5 \times 10^{-8}$ remained directionally concordant and just below the threshold ($P = 6.48 \times 10^{-8}$ for *CERS2* and $P = 5.97 \times 10^{-8}$ for *IP6K2*). We therefore agree that, while data-field 21092 contributes limited additional information, its inclusion did not materially affect the discovery findings.

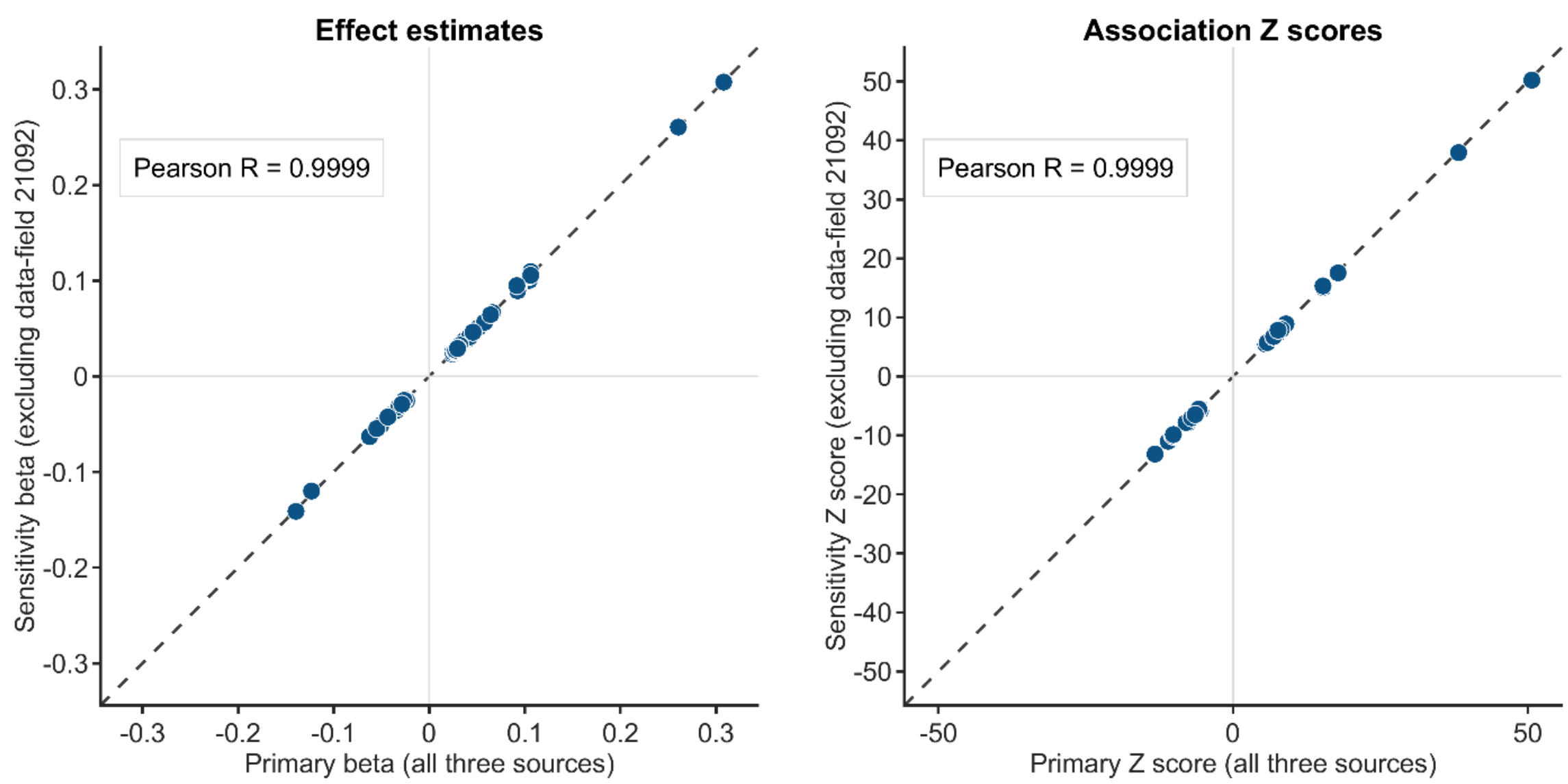

14. The abstract states that 60 loci explain 25% of the heritability, whereas the main text reports SNP-based heritability of PDFF as 25%. This wording is potentially confusing unless the manuscript clearly distinguishes variance explained by the identified loci from total SNP heritability.

We thank the reviewer and apologize for the ambiguous wording. The 25% estimate refers to the SNP-based heritability of PDFF estimated by LDSC, not to the proportion of heritability explained by the 60 lead *loci*. We have now revised the Abstract, Results and Discussion to clarify this distinction.

15. There are substantial industry relationships and patent/equity disclosures for senior authors. This does not invalidate the work, but for a translational genetics paper emphasizing PRS and biomarker implications, it reinforces the need to avoid overstatement of clinical utility.

We thank the reviewer for this comment. We have now reviewed the relevant sections and revised the wording to avoid implying clinical utility or biomarker application. The PRS, PheWAS and proteomic analyses are presented as downstream characterization of genetic susceptibility to liver triglyceride content, rather than as evidence for clinical implementation.

References: Key prior studies are cited, but the manuscript tends to benchmark novelty primarily against the authors' previous work. Broader contextualization within the existing NAFLD/MASLD GWAS literature is needed. Some loci described as "novel" may have been implicated previously in related phenotypes (e.g., ERLIN1 in Sveinbjornsson et al., 2022), and this should be more transparently addressed.

We thank the reviewer for raising this point. We agree that the novelty of the *loci* should be placed in the broader context of prior literature. In the manuscript, by "not previously reported" we explicitly mean associations with liver triglyceride content, measured by MRI-derived PDFF, rather than all MASLD-related phenotypes. We have now clarified this in the Results and added prior evidence for the 31 *loci*. The revised text now reads:

"Some of these loci had been reported previously in studies of MASLD or chronic ALT. ERLIN1 was reported in a study combining PDFF and MASLD, although its association with PDFF alone was not genome-wide significant. INSR was reported in a MASLD meta-analysis combining imaging and clinically defined phenotypes. In addition, the SLC30A10, AKNA, ERLIN1, HNF1A and NYNRIN loci were previously associated with chronic ALT in Europeans"

Clarity and context: The manuscript is well written and structured.

Reviewer #2 (Remarks to the Author):

The article reports on a UK Biobank based GWAS on liver triglyceride content with associated validation versus independent clinically manifest MASLD GWAS datasets. The analytical flow appears standard, with potential criticism mainly pertaining to phenotype definitions, confounders and reporting. The article has several interesting features and adds to current literature on MASLD-related biology.

Some points warrant consideration:

1) There is no recognition of the spectrum of ALD-MetALD-MASLD in the approach taken, in particular relevant incorporation of alcohol consumption-related variables and several important cardiometabolic risk factors (CMRFs) in the analytical approach, beyond that pertaining to the PheWAS (see also comment 8 below) presented in Figure 3. See e.g. https://www.journal-of-hepatology.eu/article/S0168-8278(25)02326-8/fulltext and co-authors on the manuscript are aware of this. The complete omission of handling alcohol as a relevant co-variable is the most significant to this reviewer.

We agree that the revised steatotic liver disease nomenclature emphasizes overlap between metabolic dysfunction, alcohol intake and cardiometabolic risk factors, and that alcohol intake should be handled explicitly. We have now added sensitivity analyses for the PRS-PDFF-GWAS associations with cirrhosis and hepatocellular carcinoma in the UK Biobank. These analyses additionally adjusted for alcohol intake, excluded individuals above the alcohol intake thresholds for metabolic dysfunction and alcohol-associated liver disease (MetALD) or alcohol-related liver disease (ALD), and applied additional endpoint exclusions for competing liver disease etiologies. The associations remained similar across these analyses. For example, per 1-s.d. increase in PRS-PDFF-GWAS, the primary association with cirrhosis was OR = 1.39/HR = 1.39; after adjustment for alcohol intake, the estimates were OR = 1.39/HR = 1.40, and after excluding individuals above the MetALD or ALD alcohol thresholds, the estimates were OR = 1.39/HR = 1.42. For hepatocellular carcinoma, the corresponding primary estimates were OR = 2.07/HR = 2.03, after alcohol adjustment OR = 2.08/HR = 2.04, and after alcohol-threshold exclusions OR = 1.92/HR = 1.88. These results are now reported in Supplementary Table 11.

Regarding cardiometabolic risk factors, all PRS endpoint analyses were already adjusted for BMI. We did not further adjust the primary models for diabetes, dyslipidemia or related cardiometabolic traits, because these factors may lie on the pathway from genetic susceptibility to liver triglyceride content to end-stage liver disease rather than acting only as confounders. We instead showed the broader cardiometabolic associations of the PRS in the PheWAS and proteomic analyses. The Results have also been revised accordingly:

"Similar associations were observed in sensitivity analyses with additional adjustment for alcohol intake, exclusion of individuals above the alcohol intake thresholds for metabolic dysfunction and alcohol-associated liver disease or alcohol-related liver disease, or additional endpoint exclusions for competing liver disease etiologies (Supplementary Table 11)."

2) The lack of generalizability to other European is hampered by UK specifics of liver disease (see e.g. PMID 30060999), enhancing the importance of point 1 – and should be mentioned in the text.

We agree that UK Biobank may not capture the full spectrum of liver disease burden across European populations. We have therefore added this point as a limitation in the Discussion:

"Generalizability may also be limited by the UK Biobank study, because liver disease burden and major risk factors may differ across European countries."

3) Robustness of the 31 claimed novel associations appears slightly opaque; for some of these loci (not easy to read from the provided materials) there is independent support for a genuine association, e.g. the overlap for 14 of the loci with previous MASLD GWAS. Exactly for how many of the remaining 17 loci there is additional support (beyond the GWS p-values in the UK Biobank query) is not clear, and reporting of these loci must acknowledge the risk of type 1 errors.
We thank the reviewer for raising this point. We agree that the support for the 31 variants not previously reported should be easier to follow in the main text. These variants were identified in the PDFF discovery meta-analysis, and their main validation was then tested in the clinically diagnosed MASLD meta-analysis. In that analysis, 14 of 31 variants reached Bonferroni significance ($P < 1.6 \times 10^{-3}$), and 22 of 31 reached nominal significance ($P < 0.05$), all with concordant directions of effect. Therefore, among the 17 variants that did not reach Bonferroni significance in MASLD, 8 still showed nominal MASLD support. We have revised the Results to list the 14 Bonferroni-significant MASLD *loci* explicitly and to state how many of the remaining *loci* had nominal MASLD support. This makes clear which *loci* had the strongest MASLD validation and which had weaker concordant support in the

MASLD meta-analysis. The revised text now reads:

"To assess the genome-wide genetic overlap between MRI-derived liver triglyceride content and clinically diagnosed MASLD, we estimated their genetic correlation using bivariate LDSC. PDFF and MASLD showed a strong genetic correlation (rg = 0.74, SE = 0.08, $P = 2.5 \times 10^{-21}$), supporting the use of MASLD as a validation phenotype for PDFF loci. Of the 31 genetic variants, 14 (45%) reached Bonferroni significance ($P < 1.6 \times 10^{-3}$) and 22 (71%) were nominally significant ($P < 0.05$), all with concordant directions of effect as compared to the discovery analyses (Supplementary Table 4A). The 14 Bonferroni-significant loci were CERS2, SLC30A10, FARP2, LEKR1, ARL15, HERPUD2, AKNA, ERLIN1, ACSL5, CCND2, HNF1A, TP53BP1, INSR and ZGPAT; among the remaining 17 loci, 8 reached nominal significance."

4) I remain puzzled about the claim that data supports "a specific inflammatory pathways in lipid-mediated hepatocyte damage". What aspects of analyzed data pertains to "hepatocyte damage"? Were transaminases considered in the analytical pipeline? Fibrosis measures?

We agree with the reviewer that the original wording was too strong. We have revised the manuscript to avoid implying that the proteomic analysis directly measured hepatocyte damage. Specifically, we removed the statement that the proteomic results provided "first genetic evidence" for a specific inflammatory pathway in lipid-mediated hepatocyte damage. The revised text now reads:

"Together, these results highlight cytokine and chemokine signaling among the plasma protein changes associated with higher genetically predicted liver triglyceride content, consistent with prior work showing that these pathways are central to hepatic inflammation and MASLD progression."

In addition, we have now tested the 60 PDFF lead variants against external GWAS summary statistics for ALT and AST. Most PDFF-increasing alleles were associated with higher ALT and AST, and these results are now reported in Supplementary Tables 7 and 8.

5) I would have expected to see a deeper cross-comparison of findings with past GWAS, beyond that pertaining to the PheWAS, similar to assessments made for MASLD, HCC and cirrhosis, i.e. including other CMRFs, and it could also have been interesting to see comparisons versus relevant blood test GWAS, including tryglycerides and transaminases.

We thank the reviewer and agree that a more comprehensive comparison with cardiometabolic traits and liver enzymes in previous studies should be included. We have now tested the 60 independent PDFF lead variants against European-ancestry GWAS summary statistics for ALT, AST, HbA1c, creatinine, systolic and diastolic blood pressure, BMI, BMI-adjusted waist-to-hip ratio, triglycerides, HDL cholesterol, LDL cholesterol, coronary artery disease, type 2 diabetes and chronic kidney disease. These results are now reported in Supplementary Table 7 and Figure 2, and the corresponding GWAS sources and proxy selection procedure have been added to the Methods. In these analyses, most PDFF-increasing alleles were associated with higher ALT and AST and with an adverse cardiometabolic profile, including higher triglycerides, HbA1c, BMI-adjusted waist-to-hip ratio, type 2 diabetes, coronary artery disease and blood pressure, and lower HDL cholesterol. We also found that PDFF-increasing alleles were often associated with lower BMI but higher BMI-adjusted waist-to-hip ratio and type 2 diabetes risk. We additionally examined trans-ancestry ALT associations in summary statistics from MVP and BioBank Japan. The revised text now reads:

"We next tested the association of the 60 independent PDFF lead variants with liver enzymes and cardiometabolic traits using European-ancestry GWAS summary statistics for ALT, AST, HbA1c,

creatinine, systolic and diastolic blood pressure, BMI, WHRadjBMI, triglycerides, HDL cholesterol, LDL cholesterol, coronary artery disease, type 2 diabetes and chronic kidney disease (Supplementary Table 7 and Figure 2). Most PDFF-increasing alleles were associated with higher ALT and AST and with higher triglycerides, HbA1c, WHRadjBMI, type 2 diabetes, coronary artery disease and blood pressure, and lower HDL cholesterol. PDFF-increasing alleles were often associated with lower BMI (20/23), but higher WHRadjBMI (24/26) and type 2 diabetes risk (33/37) at Bonferroni $P < 0.05/60$, showing that the allele increasing liver triglyceride content was frequently not the BMI-increasing allele. Among the 31 newly identified variants, those replicated in our MASLD meta-analysis included several loci with prior evidence, including CCND2 and ARL15 for type 2 diabetes, CERS2 for kidney function, and BCL2 for body fat distribution. Consistent with our previous work on partitioning the variants based on their lipid retention pattern, triglyceride-increasing loci showed higher coronary artery disease risk (10/10) and type 2 diabetes risk (21/23), whereas triglyceride-lowering loci more often showed lower coronary artery disease risk (3/4) but still higher type 2 diabetes risk (5/6).
We further examined ALT associations in ancestry-specific MVP summary statistics and BioBank Japan (Supplementary Table 8 and Supplementary Fig. 3). Forty-two loci were associated with ALT in Europeans ($P < 0.05/60$), all concordant with the PDFF discovery effect. In African and admixed American ancestries, the AKNA, ERLIN1 and COBLL1 loci were associated with ALT at $P < 0.05/60$, as were the GPAM and TRIB1 loci in admixed American and Japanese ancestries. Overall, 37 loci showed nominal ALT associations and 16 were associated at $P < 0.05/60$ in non-European ancestries, all with concordant directions."

6) I fully understand and agree to the exclusion of viral hepatitis (in both analyses? – page 13 – how many?) but would have found analyses on specific features to that end useful to expand on the topic into this subpopulation.

We agree that analyses within participants with chronic viral hepatitis would be of interest. However, this subgroup was small in the present analysis (n = 450), and subgroup analyses of cirrhosis or HCC within this group would therefore be underpowered. In the PheWAS, PRS-PDFF-GWAS was not associated with viral hepatitis phecodes, including viral hepatitis overall, viral hepatitis B, or viral hepatitis C (Supplementary Table 12). We have clarified in the Methods that chronic viral hepatitis was excluded from both the cirrhosis and HCC analyses and added the number of excluded individuals.

7) I am not sure Figure 2 contains sufficient information over and above that presented in the text to justify a separate display item; clarifications to point 3 above would be more useful (e.g. table).

We agree with the reviewer and have therefore removed Figure 2. The PRS decile analysis is still reported in the Results, and the full decile estimates for cirrhosis and HCC, using either decile 1 or the middle deciles as the reference group, are provided in Supplementary Table 9 and Supplementary Fig. 4.

8) In Figure 3, under "mental disorders", I find "Alcoholic liver damage". How come? To this reviewer, this is unacceptable (and the term "alcoholic" is no longer used in relation to liver disease, as co-authors of the manuscript would be aware).

We thank the reviewer for pointing this out. We agree that displaying this phenotype under mental disorders was misleading, and we have moved it to digestive disorders in Figure 3 and Supplementary Table 12. We also agree that "alcoholic" is no longer the preferred clinical terminology for alcohol-associated liver disease. However, this phenotype name was not introduced by us. It comes from the source phecode/Pan-UKB phenotype label for phecode 317.11, "Alcoholic liver damage", which is defined by ICD-10 K70 alcohol-related liver disease codes. Phecode-based resources are widely used for EHR-based PheWAS and biobank analyses (PMID: 20335276 and

31553307), including Pan-UKB (PMID: 40968291), All of Us through phecodeX (PMID: 37930895, 38374255), or FinnGen (PMID: 36653562). We therefore retained the source phenotype name to keep the result traceable to the phenotype used in the analysis, while correcting its display category from mental disorders to digestive disorders.

Reviewer #2 (Remarks to the Author):

The authors have made some effort in responding to reviewer requests. For alcohol-intake (my point #1), as a key co-variable, also for the individual associations, Supplementary Table 11 (btw: Supplementary Tables remain poorly formatted) does not respond to the request as pertaining to the individually claimed/reported associations, only to findings on a global level. Given the many marginal (individual variants) associations claimed (amongst the total claimed 31 newly identified variants), I would expect to be able to see how many of the individual associations are significantly driven by alcohol-intake (versus other metabolic pathways) towards facilitating liver triglyceride accumulation. Further, to respond to my criticism point #2, by including a mere/single sentence in the discussion, largely neglects the problem of multiple claimed novel loci (some marginal, some non-validated) that still appear to rely on analysis of the single UK-based cohort (as also pointed out by reviewer #1). These issues are not yet sufficiently clear to the reader.

We thank the reviewer for clarifying this point. We agree that Supplementary Table 11 (now S12) addressed the PRS endpoint analyses and did not report alcohol-adjusted results for the individual GWAS loci. We therefore repeated association testing for the 60 PDFF lead variants after further adjustment for alcohol intake. The results are provided in the new Supplementary Table 4 and summarized below together with the primary GWAS results. The corresponding z-score comparison is shown in the figure below. Effect estimates were highly similar to the primary GWAS (Pearson r = 0.999), and all 60 lead variants had the same direction of effect after alcohol adjustment. Fifty-eight variants remained genome-wide significant. The two *loci* just above the genome-wide significance threshold after alcohol adjustment were *SSR2* ($P = 1.3 \times 10^{-7}$) and *ARL15* ($P = 7.5 \times 10^{-8}$). We have added this analysis to the Results and Methods. The revised Results now reads:

"After additional adjustment for alcohol intake, effect estimates were highly similar to the primary GWAS (Pearson r = 0.999), and all 60 lead variants had directions of effect concordant with the primary GWAS. Fifty-eight variants remained genome-wide significant, whereas the SSR2 ($P = 1.3 \times 10^{-7}$) and ARL15 ($P = 7.5 \times 10^{-8}$) loci were just above the genome-wide significance threshold (Supplementary Table 4)."

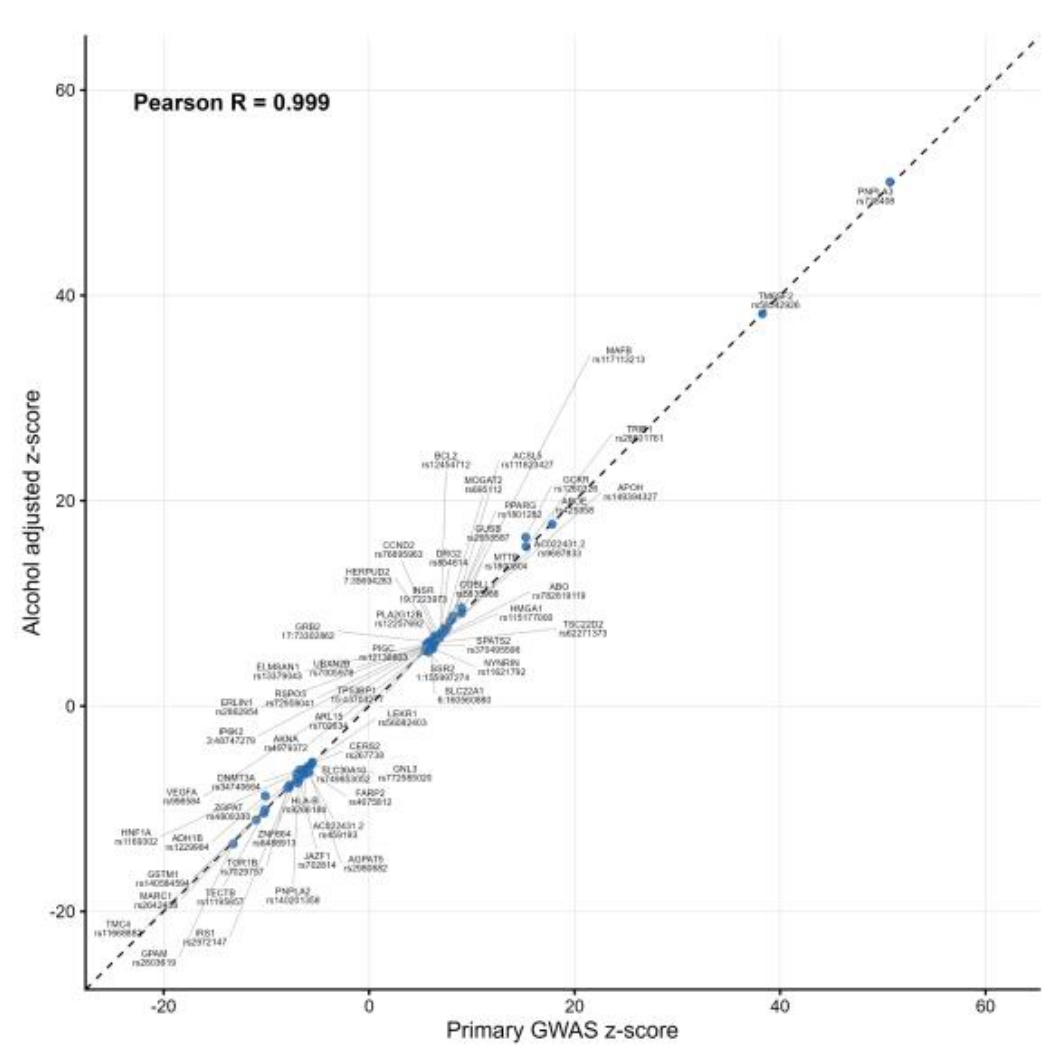

Locus (effect allele)	Primary GWAS (n = 71488)			Alcohol adjusted (n = 70560)		
	Beta	SE	P	Beta	SE	P
GSTM1, rs140584594 (A)	-0.037	0.005	9.54E-15	-0.036	0.005	1.5E-14
CERS2, rs267738 (T)	-0.028	0.005	2.76E-08	-0.028	0.005	2.2E-08
SSR2, 1:155997274_CTATT_C (CTATT)	0.025	0.004	6.61E-09	0.023	0.004	1.3E-07
PIGC, rs12138803 (T)	0.028	0.005	2.50E-09	0.029	0.005	1.1E-09
SLC30A10, rs749853052 (T)	-0.028	0.005	2.37E-09	-0.027	0.005	3.2E-09
MARC1, rs2642438 (A)	-0.051	0.005	4.71E-28	-0.051	0.005	1.5E-28
DNMT3A, rs34740664 (CT)	-0.030	0.004	1.65E-11	-0.027	0.004	5.9E-10
GCKR, rs1260326 (T)	0.066	0.004	1.74E-52	0.071	0.004	8.9E-61
COBLL1, rs5835988 (T)	0.039	0.004	2.39E-19	0.041	0.004	8.9E-22
IRS1, rs2972147 (T)	-0.034	0.004	9.33E-15	-0.035	0.004	3.0E-15
FARP2, rs4675812 (A)	-0.026	0.004	6.96E-10	-0.026	0.004	1.1E-09
PPARG, rs1801282 (C)	0.051	0.006	3.17E-15	0.054	0.006	1.2E-16
IP6K2, 3:48747279_AAATT_A (A)	0.104	0.018	1.44E-08	0.106	0.018	8.0E-09
GNL3, rs772585020 (A)	-0.024	0.004	1.48E-08	-0.024	0.004	9.7E-09
TSC22D2, rs62271373 (A)	0.059	0.009	1.26E-10	0.056	0.009	5.9E-10
LEKR1, rs56082403 (T)	-0.024	0.004	3.45E-08	-0.024	0.004	4.9E-08
ADH1B, rs1229984 (T)	-0.140	0.014	5.07E-24	-0.120	0.014	2.2E-18
MTTP, rs1800804 (T)	0.044	0.005	2.54E-19	0.044	0.005	1.6E-19
ARL15, rs702634 (A)	0.025	0.005	4.30E-08	0.025	0.005	7.5E-08
AC022431.2, rs459193 (A)	-0.029	0.005	1.89E-09	-0.031	0.005	2.5E-10
AC022431.2, rs9687833 (A)	0.036	0.005	3.39E-12	0.036	0.005	7.6E-12
HLA-B, rs9266184 (T)	-0.029	0.005	3.68E-10	-0.031	0.005	2.6E-11
HMGA1, rs115177000 (A)	0.065	0.010	2.70E-10	0.064	0.010	3.7E-10
VEGFA, rs998584 (A)	0.024	0.004	1.13E-08	0.024	0.004	7.2E-09
RSPO3, rs72959041 (A)	0.058	0.010	4.53E-09	0.059	0.010	2.0E-09
SLC22A1, 6:160560880_CATG_C (CATG)	0.032	0.005	3.08E-09	0.032	0.005	4.7E-09
JAZF1, rs702814 (T)	-0.029	0.004	8.90E-12	-0.030	0.004	7.0E-13
HERPUD2, 7:35694283_CT_C (CT)	0.028	0.005	2.43E-10	0.031	0.004	9.4E-12
GUSB, rs2658587 (A)	0.031	0.004	2.68E-13	0.032	0.004	2.8E-14
AGPAT5, rs2980682 (A)	-0.026	0.004	5.70E-09	-0.029	0.004	1.1E-10
UBXN2B, rs7005978 (A)	0.027	0.004	1.18E-09	0.027	0.004	1.0E-09
TRIB1, rs28601761 (C)	0.066	0.004	9.83E-53	0.067	0.004	1.5E-54
AKNA, rs4979372 (T)	0.024	0.004	1.44E-08	0.024	0.004	2.7E-08
TOR1B, rs7029757 (A)	-0.058	0.007	1.77E-15	-0.058	0.007	1.2E-15
ABO, rs782819119 (A)	0.028	0.005	1.97E-09	0.029	0.005	5.3E-10
PLA2G12B, rs12257692 (T)	0.064	0.010	4.40E-10	0.066	0.010	1.4E-10
ERLIN1, rs2862954 (T)	0.025	0.004	4.93E-09	0.025	0.004	2.0E-09
GPAM, rs2803619 (C)	-0.062	0.005	6.25E-40	-0.063	0.005	4.9E-41
TECTB, rs11195857 (A)	-0.055	0.005	1.37E-24	-0.056	0.005	1.9E-25
ACSL5, rs111823427 (A)	0.093	0.011	3.33E-16	0.100	0.011	1.5E-18
PNPLA2, rs140201358 (C)	-0.123	0.018	4.27E-12	-0.133	0.018	6.3E-14
MOGAT2, rs695112 (A)	0.042	0.006	5.66E-13	0.043	0.006	1.2E-13
CCND2, rs76895963 (T)	0.106	0.016	6.73E-11	0.110	0.016	1.5E-11
SPATS2, rs370495596 (CA)	0.046	0.007	5.27E-10	0.043	0.007	5.8E-09
HNF1A, rs1169302 (T)	-0.030	0.004	1.95E-12	-0.028	0.004	5.5E-11
ZNF664, rs6488913 (C)	-0.031	0.004	1.85E-12	-0.032	0.004	1.1E-12
NYNRIN, rs11621792 (T)	0.026	0.004	5.26E-10	0.024	0.004	2.2E-08
ELMSAN1, rs13379043 (T)	0.027	0.005	2.25E-08	0.029	0.005	1.3E-09
TP53BP1, 15:43703277_AT_A (A)	0.105	0.019	3.66E-08	0.106	0.019	2.1E-08
DRG2, rs854814 (A)	0.032	0.005	4.37E-12	0.033	0.005	1.8E-12
APOH, rs149394327 (C)	0.092	0.013	2.44E-13	0.091	0.013	5.0E-13
GRB2, 17:73302862_TA_T (T)	0.028	0.005	6.00E-09	0.030	0.005	3.9E-10
BCL2, rs12454712 (T)	0.030	0.004	4.79E-12	0.030	0.004	2.1E-12
INSR, 19:7223973_TTTG_T (T)	0.030	0.004	7.22E-12	0.029	0.004	3.1E-11
TM6SF2, rs58542926 (T)	0.308	0.008	0.00E+00	0.307	0.008	0.0E+00
APOE, rs429358 (T)	0.106	0.006	5.16E-71	0.106	0.006	3.3E-70
TMC4, rs11668882 (T)	-0.043	0.004	4.09E-24	-0.043	0.004	5.4E-24
MAFB, rs117113213 (A)	0.092	0.012	2.59E-14	0.092	0.012	1.9E-14
ZGPAT, rs4809330 (A)	-0.029	0.004	1.35E-10	-0.029	0.004	9.2E-11
PNPLA3, rs738408 (T)	0.261	0.005	0.00E+00	0.262	0.005	0.0E+00

We also apologize if the PRS sensitivity table was difficult to inspect in the previous submission. Supplementary Table 11 was submitted as an Excel sheet, but it appears to have been displayed to the reviewer in a less readable format. For clarity, this table is now Supplementary Table 12 and is reported below. This table addresses the PRS endpoint analyses, whereas the new Supplementary Table 4 addresses the individual GWAS lead variants.

Supplementary Table 12. Sensitivity analyses for association of PRS-PDFF-GWAS with end-stage liver disease after accounting for alcohol intake and competing liver disease etiologies in the UK Biobank. Associations between the genome-wide PRS for liver fat content (PDFF) and cirrhosis or hepatocellular carcinoma (HCC) were tested using logistic (OR, odds ratio) or Cox proportional hazards (HR, hazard ratio) regression models. Primary refers to the endpoint definitions used in Supplementary Table 11, in which chronic viral hepatitis was excluded. Restricted refers to sensitivity analyses in which cholestatic liver disease (ICD-10 K74.3, K74.4 and K74.5), autoimmune hepatitis (K75.4), alcohol-related liver disease (K70.0, K70.1, K70.2, K70.3, K70.4 and K70.9), and disorders of copper or iron metabolism (E83.0 and E83.1) were additionally excluded in case-control analyses and censored at diagnosis in prospective analyses. Base indicates no additional alcohol adjustment or alcohol-based exclusion. Adjusted indicates additional adjustment for alcohol intake. ALD-range excluded indicates exclusion of individuals with alcohol intake above 60 g/day in men or 50 g/day in women. MetALD/ALD excluded indicates exclusion of individuals with alcohol intake above 30 g/day in men or 20 g/day in women. All analyses were adjusted for BMI, age, sex, age×sex, age² and age²×sex, the first 10 genomic principal components, and array batch. All association analyses were performed after excluding individuals with available PDFF (n = 71,488). OR and HR represent the effect per 1-s.d. increase in PRS-PDFF-GWAS and are shown with 95% confidence intervals. P-values are two-sided and were not corrected for multiple testing. Abbreviations: PRS, polygenic risk score; HCC, hepatocellular carcinoma; ALD, alcohol-related liver disease; MetALD, metabolic dysfunction and alcohol-associated liver disease.

Pheno	Endpoint	Alcohol analysis	Cases/N	OR (95% CI), P	Events/N	HR (95% CI), P
Cirrhosis	Primary	Base	2854/303618	1.39 [1.34, 1.44], 1.21E-65	2366/300688	1.39 [1.34, 1.45], 3.81E-57
Cirrhosis	Primary	Adjusted	2822/302265	1.39 [1.34, 1.44], 3.56E-65	2347/299374	1.40 [1.35, 1.46], 4.90E-59
Cirrhosis	Primary	ALD-range excluded	2467/294066	1.40 [1.35, 1.46], 2.24E-61	2018/291309	1.41 [1.35, 1.47], 1.31E-52
Cirrhosis	Primary	MetALD/ALD excluded	1921/242963	1.39 [1.33, 1.46], 1.44E-45	1536/240623	1.42 [1.35, 1.49], 1.63E-41
Cirrhosis	Restricted	Base	1711/300518	1.32 [1.26, 1.39], 1.98E-29	1964/300366	1.39 [1.33, 1.45], 3.15E-47
Cirrhosis	Restricted	Adjusted	1698/299204	1.32 [1.26, 1.39], 1.49E-29	1948/299059	1.40 [1.33, 1.46], 1.07E-47
Cirrhosis	Restricted	ALD-range excluded	1619/291454	1.35 [1.28, 1.41], 9.95E-32	1771/291009	1.41 [1.34, 1.47], 9.69E-46
Cirrhosis	Restricted	MetALD/ALD excluded	1358/241077	1.35 [1.28, 1.42], 6.77E-27	1423/240400	1.40 [1.33, 1.48], 2.84E-36
HCC	Primary	Base	414/303618	2.07 [1.89, 2.28], 2.52E-52	409/303577	2.03 [1.85, 2.23], 5.87E-49
HCC	Primary	Adjusted	412/302265	2.08 [1.89, 2.29], 1.08E-52	407/302223	2.04 [1.86, 2.24], 2.27E-49
HCC	Primary	ALD-range excluded	367/294066	2.03 [1.83, 2.24], 8.54E-44	361/294009	1.97 [1.78, 2.18], 7.89E-40
HCC	Primary	MetALD/ALD excluded	286/242963	1.92 [1.71, 2.15], 2.14E-29	280/242922	1.88 [1.67, 2.10], 5.09E-27
HCC	Restricted	Base	286/300518	1.79 [1.60, 2.01], 1.92E-23	330/302868	1.92 [1.72, 2.13], 1.94E-33
HCC	Restricted	Adjusted	285/299204	1.80 [1.60, 2.01], 1.31E-23	329/301528	1.92 [1.73, 2.14], 9.15E-34
HCC	Restricted	ALD-range excluded	272/291454	1.81 [1.61, 2.04], 3.29E-23	302/293350	1.88 [1.68, 2.10], 7.41E-29
HCC	Restricted	MetALD/ALD excluded	221/241077	1.78 [1.56, 2.03], 3.53E-18	237/242390	1.80 [1.59, 2.04], 3.04E-20

Regarding the reviewer's second point, we agree that the PDFF discovery GWAS was based on UK Biobank imaging and that this should be stated clearly as a limitation. However, we would like to clarify that the external validation of the newly identified loci was not based on a single UK cohort. The MASLD validation meta-analysis included deCODE, Intermountain, FinnGen, MVP and UK Biobank, with individuals with available PDFF measurements excluded from the UK Biobank component. As described in our response to Reviewer 1, exclusion of UK Biobank from the MASLD meta-analysis did not materially change the validation results. We also assessed the comparability of the MASLD validation phenotype across studies: PDFF and clinically diagnosed MASLD showed strong genome-wide genetic correlation ($r_g = 0.74$, SE = 0.08, $P = 2.5 \times 10^{-21}$), and the main MASLD sources were highly genetically correlated with each other. These analyses support genetic concordance across the MASLD sources despite differences in clinical definitions and liver disease burden.

To make the remaining limitation clearer to the reader, we have now expanded the Discussion. The revised text now reads:

"In addition, the MRI-PDFF discovery analysis was based on UK Biobank imaging and therefore does not provide replication in an independent imaging cohort. External support for the newly identified loci also varied: the MASLD meta-analysis supported 22 of the 31 loci at nominal significance, including 14 after Bonferroni correction, whereas the remaining loci should be interpreted as PDFF discovery associations until replicated in larger external imaging studies."
